# Microbial genetic screen identifies bacterial genes that compromise *Caenorhabditis elegans* reproductive fitness

**DOI:** 10.1128/msystems.01698-25

**Published:** 2026-05-07

**Authors:** Ziling Yang, Huigui Guo, Yudong Zhang, Xinzhou Jia, Yalun Wu, Tao Zhu, Ying Li, Jinyue Wang, Dianshuang Zhou, Zuobin Zhu

**Affiliations:** 1 Key Laboratory of Genetic Foundation and Clinical Application, Department of Genetics, Xuzhou Medical University, Jiangsu Engineering Center for Precision Diagnosis and Treatment Research of Polygenic Diseases, Xuzhou, China; 2 School of the First Clinical Medicine, Xuzhou Medical University 38044 , Xuzhou, China; The Pennsylvania State University, State College, Pennsylvania, USA; Vietnam National University of Forestry, Hanoi, Vietnam

**Keywords:** microbial genetic variation, reproductive fitness, oxidative phosphorylation, multi-omics

## Abstract

The mechanisms by which microbial genetic variation governs host reproductive fitness remain to be determined. Defining the causal relationship between specific microbial genes and host reproduction not only facilitates understanding of the microbe–host interaction mechanism but also provides new insights for reproductive health interventions. However, the complexity of the gut microbial community poses a challenge, and related research has been constrained by a lack of systematic approaches. In the present study, we utilized the *Escherichia coli* Keio collection that comprises 3,467 strains to screen for three mutant strains *ΔcrcB*, *ΔpurE*, and *ΔyojI* that markedly inhibit the reproductive fitness of *Caenorhabditis elegans*. These mutants reduce offspring number and prolong the time to first reproduction, specifically inhibiting spermatogenesis, while exerting no impact on locomotor function or lifespan. Multi-omics analyses revealed that these gene deletions trigger bacterial metabolic remodeling, characterized by the accumulation of cytidine diphosphate-diacylglycerol (CDP-DG) and disturbances in nucleotide and glycerophospholipid metabolism. These changes further regulate oxidative phosphorylation (with gene upregulation) and cell cycle pathways (with gene downregulation) in the host nematode, thereby inducing a “high metabolism, low proliferation” state and ultimately reducing reproductive fitness. This study delineates the one-to-one correspondence between gene deletions in *Escherichia coli* and alterations in nematode reproduction at the single-gene level, providing a novel framework for dissecting the molecular basis of host reproductive disorders from a microbial genetics perspective.

**IMPORTANCE:**

This study identifies a direct causal link between a specific bacterial gene and host reproductive fitness. Systematic screening of *Escherichia coli* mutants showed that deleting one gene severely impairs *Caenorhabditis elegans* reproduction. Using a controllable model, we achieved precise microbe gene–host reproductive phenotype mapping, revealing the mechanism: bacterial gene deficiency induces host “high metabolism, low proliferation” to reduce fertility. It offers a new genetic perspective for understanding microbe-mediated reproductive disorders and a framework for dissecting single-gene microbe–host interactions and developing targeted interventions.

## INTRODUCTION

The intestinal microbiota and the host maintain a complex interactive relationship, which has been proven to be a key factor regulating the host’s metabolism, immunity, neurobehavior, and even reproductive processes (1–4). In recent years, a number of studies have revealed the important role of the overall structure of the intestinal microbial community and its metabolites in influencing the host’s reproductive health. For instance, specific microbial groups can interfere with the host’s hormonal signaling pathways, epigenetic modifications, or embryonic development programs through their metabolites, thereby regulating reproductive output (5–7). However, most of these studies are limited to the level of “entire community” or “multi-microbial synergy,” making it difficult to clarify the function of individual microbial genes and their specific role in host reproductive regulation.

During the long-term co-evolution of microorganisms and hosts, a close functional association has been formed, and the genetic variation of microorganisms will inevitably affect the way they interact with hosts (8–10). Theoretically, identifying microbial genetic variations that impair host fecundity is of great significance for understanding the microbial causes of reproductive disorders, developing novel diagnostic methods, and formulating probiotic intervention strategies. However, due to the high complexity and significant individual differences of the intestinal microbial system, it is extremely challenging to accurately dissect the correspondence between “single microbial genotype and host reproductive phenotype” *in vivo*.

The *Escherichia coli* single-gene knockout library (Keio collection [11]) provides a powerful tool for addressing this challenge. This library covers single-gene knockout mutants of nearly all non-essential genes in the *E. coli* K-12 genome, which is equivalent to centrally simulating the genetic diversity that requires long-term accumulation in the natural evolution process under laboratory conditions. By combining with *Caenorhabditis elegans*—a model organism with a simple intestinal microbiota structure and a well-established genetic manipulation system (12)—it is possible to achieve high-throughput screening of microbial genetic variations that affect host reproductive fitness under the premise of strictly controlling the microbial background.

In this study, the Keio collection of *E. coli* was employed, and *C. elegans* was used as the host model to systematically assess the impacts of 3,467 single-gene knockout strains on the reproductive ability of nematodes. We identified three mutant strains—*ΔcrcB*, *ΔpurE*, and *ΔyojI*—that significantly impair *C. elegans* reproduction and elucidated their potential mechanisms of action through integrated multi-omics analyses. This study not only establishes a direct link between microbial genetic perturbations and host reproductive phenotypes at the single-gene resolution but also underscores the utility of controlled microbe–host systems in dissecting complex biological processes. Moreover, it provides a novel theoretical framework and research paradigm for investigating reproductive regulation through the lens of microbial genetics.

## MATERIALS AND METHODS

### Experimental subject: *C. elegans*

The wild-type strain N2 of *C. elegans*, used as the host model in this study, was provided by the Caenorhabditis Genetics Center at the University of Minnesota, USA. For routine maintenance, *C. elegans* was cultured on nematode growth medium (NGM) agar plates supplemented with *E. coli* OP50 (as a food source) at a constant temperature of 20°C.

### Bacterial strains: *E. coli* single-gene knockout strains

All *E. coli* single-gene knockout strains employed in the experiment were derived from the Keio collection. Prior to use, *E. coli* strains were cultured with shaking in Luria-Bertani (LB) medium at 37°C and 200 rpm until reaching the logarithmic growth phase (OD_600_ of 0.6–0.8). The bacterial culture was then pelleted by centrifugation at 7,000 rpm for 5 min; after discarding the supernatant, the bacterial pellet was resuspended in 1,000 μL of fresh LB medium, and 100 μL of the resuspended culture was transferred onto NGM plates using a pipette for subsequent *C. elegans* feeding and experimental assays.

### Determination of reproductive fitness

Synchronization was achieved via the alkaline sodium hypochlorite lysis method, and the nematodes were then incubated in M9 buffer overnight to grow into L1-stage larvae. L1-stage larvae were inoculated onto NGM plates pre-coated with bacterial suspension and cultured in a 20°C incubator for 24 h to develop into L4-stage larvae. Two methods were used for the determination of reproductive fitness.

(i) For high-throughput screening, individual L4-stage nematodes were transferred to NGM plates (30 mm in diameter) inoculated with respective mutant strains. After culturing at 20°C for 4 days, the number of progeny nematodes was automatically counted using Worm Studio (13)—an image analysis software independently developed by our research group—to improve statistical efficiency.

(ii) For temporal reproductive pattern analysis, to accurately evaluate the progeny production dynamics throughout the entire reproductive cycle, individual L4-stage nematodes were sequentially transferred to NGM plates inoculated with respective mutant strains, with plates replaced every 24 h until the individuals ceased egg laying. The original plates were further cultured at 20°C for 72 h, and counting was performed after the progeny developed into the L4 stage or early adult stage. This protocol prevents confusion between parental and progeny nematodes and provides accurate data on the number of progeny produced per day.

### Bacterial heat inactivation assay

To determine whether the reproductive inhibitory effect of the mutant strains required live bacterial metabolism, *E. coli* strains (BW25113, *ΔcrcB*, *ΔpurE*, and *ΔyojI*) were subjected to heat inactivation. Bacterial cultures were grown to logarithmic phase as described above. The cultures were then placed in a 65°C water bath for 30 min. After heat treatment, 100 μL of the inactivated bacterial suspension was spread onto LB agar plates and incubated overnight at 37°C to verify complete inactivation (no colony formation). Only batches showing no growth were used for subsequent *C. elegans* feeding assays. The inactivated bacteria were then spread onto NGM plates, and *C. elegans* reproductive fitness was assessed as described in “Determination of reproductive fitness.” above.

### Lifespan assay

For the lifespan assay of *C. elegans*, synchronized individuals precisely at the young adult stage (day 1 of adulthood) were selected and transferred to NGM plates with four different bacterial food conditions: BW25113 wild-type group, *ΔcrcB* group, *ΔpurE* group, and *ΔyojI* group. Subsequently, their survival status was determined daily by observing pharyngeal pumping and responses to mechanical stimulation using a platinum wire. Individuals were judged as dead if they showed no pharyngeal pumping and no response to stimulation.

### Locomotor ability assay

For the assessment of locomotor ability, a quantitative head thrash frequency assay was adopted. Synchronized *C. elegans* were cultured under respective experimental conditions until reaching the young adult stage (day 1), after which individual nematodes were transferred to fresh NGM plates without bacterial food. Only one nematode was placed on each plate, and 10 μL of sterile M9 buffer was added. Following a 1 min acclimation period at 20°C, a high-resolution charge-coupled device (CCD) camera was used to record the nematodes’ movement in the liquid environment for 30 s. The number of complete head thrashes (from left to right) was calculated precisely.

### DAPI staining of *C. elegans* germ cells

Ten synchronized hermaphroditic *C. elegans* at the L4 stage (20 h post-synchronization) were selected and transferred to a microscope slide containing 10 μL of M9 buffer. The worms were washed three times with 90% ethanol. The staining procedure was performed under light-protected conditions: 10 μL of 0.5 μg/mL 4′,6-diamidino-2-phenylindole (DAPI) stain was added, and after covering with a coverslip, the samples were incubated in the dark for 5 min. Samples were observed using a conventional fluorescence microscope. Under a 40× objective lens, sperm nuclei exhibited characteristic blue fluorescence, appearing as granular structures with uniform size and tight arrangement.

### RNA-seq analysis of *C. elegans*

A comprehensive experimental design was adopted for transcriptome sequencing, including control samples fed with BW25113 and three experimental groups fed with *ΔcrcB*, *ΔpurE*, and *ΔyojI*, respectively. Each group contained six biological replicates, resulting in a total of 24 samples. The clean data of each sample reached over 6.01 Gb (BioProject: F21FTSECWLJ1283_NEMyjzwN).

First, adapter sequences, low-quality reads, sequences with a high rate of ambiguous bases, and excessively short sequences were filtered out from the raw sequencing data. Subsequently, clean reads were aligned to the reference genome (NCBI: GCF_000002985.6_WBcel235) using HiSat2 (14), and the expression levels of genes and transcripts were quantified separately using the RSEM (15) software. DESeq2 (16) was used to perform inter-sample gene expression difference analysis for multi-sample (≥2) projects, identifying differentially expressed genes (DEGs) between samples to further investigate the functions of these DEGs. Genes with a |log₂FC| > 1 and *P* < 0.05 were considered to have significantly different expression. Finally, Kyoto Encyclopedia of Genes and Genomes (KEGG) enrichment analysis and Gene Set Enrichment Analysis (GSEA) (17) were conducted to analyze these DEGs.

### Proteomic sequencing of *E. coli*

For proteomic and metabolomic analyses, *E. coli* strains (BW25113, *ΔcrcB*, *ΔpurE*, and *ΔyojI*) were cultured in LB medium to logarithmic phase (OD_600_ of 0.6–0.8). Bacterial cells were harvested by centrifugation at 7,000 rpm for 5 min at 4°C. The supernatant was discarded, and the bacterial pellet was washed three times with ice-cold phosphate-buffered saline. The pellet was then flash-frozen in liquid nitrogen and stored at −80°C until further processing. Six biological replicates were collected per group. The samples were taken out in a frozen state, and protein lysis buffer was added. After tissue grinding, sonication, and centrifugation, the supernatant was collected, and protein quantification was performed using the bicinchoninic acid (BCA) method. A 100 μg aliquot of the protein solution was subjected to reduction and alkylation treatments with tris(2-carboxyethyl)phosphine and iodoacetamide sequentially, followed by protein precipitation with cold acetone. After the precipitate was dissolved, it was enzymatically digested overnight at 37°C using trypsin. The resulting peptides were desalted using an hydrophilic-lipophilic balance (HLB) column, quantified, and then subjected to data-independent acquisition mass spectrometry detection. Mass spectrometry analysis was conducted on an Orbitrap Astral mass spectrometer. Peptides were separated using a Vanquish Neo liquid chromatography system and a µPAC column (75 μm × 5.5 cm), with the mobile phase consisting of water and acetonitrile containing 0.1% formic acid, and the separation duration was 8 min. The ion source voltage was set to 1.5 kV, and the scanning range was 100–1700 *m*/*z*.

Raw data were analyzed using Spectronaut 19 software, with the UniProt database used for database searching. The false discovery rate (FDR) thresholds for both proteins and peptides were set to ≤0.01. Differentially expressed proteins (DEPs) were screened based on the criteria of *P* < 0.05 and a fold change greater than 1. Functional annotation, pathway enrichment analysis, and Cytoscape interaction network analysis of these differentially expressed proteins were performed using the Gene Ontology (GO) database, KEGG database, and String platform, respectively.

### Untargeted metabolomics by LC-MS/MS

For metabolomic analysis of *C. elegans*, synchronized L1-stage larvae were fed the respective *E. coli* strains (BW25113, *ΔcrcB*, *ΔpurE*, and *ΔyojI*) on NGM plates at 20°C. Young adult worms (approximately 24 h post-L4 stage) were collected. Worms were washed three times with M9 buffer to remove residual bacteria, allowed to settle by gravity, and the supernatant was aspirated. The clean worm pellets were flash-frozen in liquid nitrogen and stored at −80°C. Each group collected six biological replicate samples, and the total volume of each sample was approximately 0.5 mL. The experiment was performed using a Thermo Fisher UHPLC-Q Exactive HF-X system coupled with an HSS T3 column (100 mm × 2.1 mm, 1.8 μm). Mobile phase A consisted of water-acetonitrile (95:5) containing 0.1% formic acid, and mobile phase B was acetonitrile-isopropanol-water (47.5:47.5:5) containing 0.1% formic acid. For the positive ion mode gradient, phase B increased from 0% to 100% (0–6.3 min); the negative ion mode gradient was similar. The flow rate was 0.4 mL/min, and the column temperature was 40°C. Mass spectrometry scanning was performed in a positive/negative ion switching mode with a mass range of 70–1,050 *m*/*z*, a primary resolution of 60,000, a secondary resolution of 7,500, and data acquisition in data-dependent acquisition (DDA) mode.

After sample preparation, quality control (QC) samples were inserted (once every 5–15 samples) to monitor stability. Data were processed using Progenesis QI, with missing values imputed using the 80% rule, followed by total normalization and log10 transformation. Variables with a QC relative standard deviation (RSD) < 30% were filtered. Differential metabolites were identified using orthogonal projections to latent structures discriminant analysis (OPLS-DA) (Vip > 1) and Student’s *t*-test (*P* < 0.05). KEGG pathway enrichment analysis was performed using Fisher’s exact test via Python’s scipy.stats package. All analyses were completed on the Majorbio Cloud platform (18).

### Statistical analysis

Reproductive fitness, lifespan, and locomotor activity were all analyzed using GraphPad Prism version 9.4.1. Dunnett’s test was applied to compare all treatment groups with the control group. *P < 0.05, **P < 0.01, ***P < 0.001, and ****P < 0.0001; “ns” indicates no statistically significant effect. The survival curves of *C. elegans* were analyzed using the log-rank (Mantel-Cox) test.

## RESULTS

### High-throughput screen identifies *E. coli* single-gene deletion mutants that inhibit *C. elegans* reproduction

**Fig 1:**
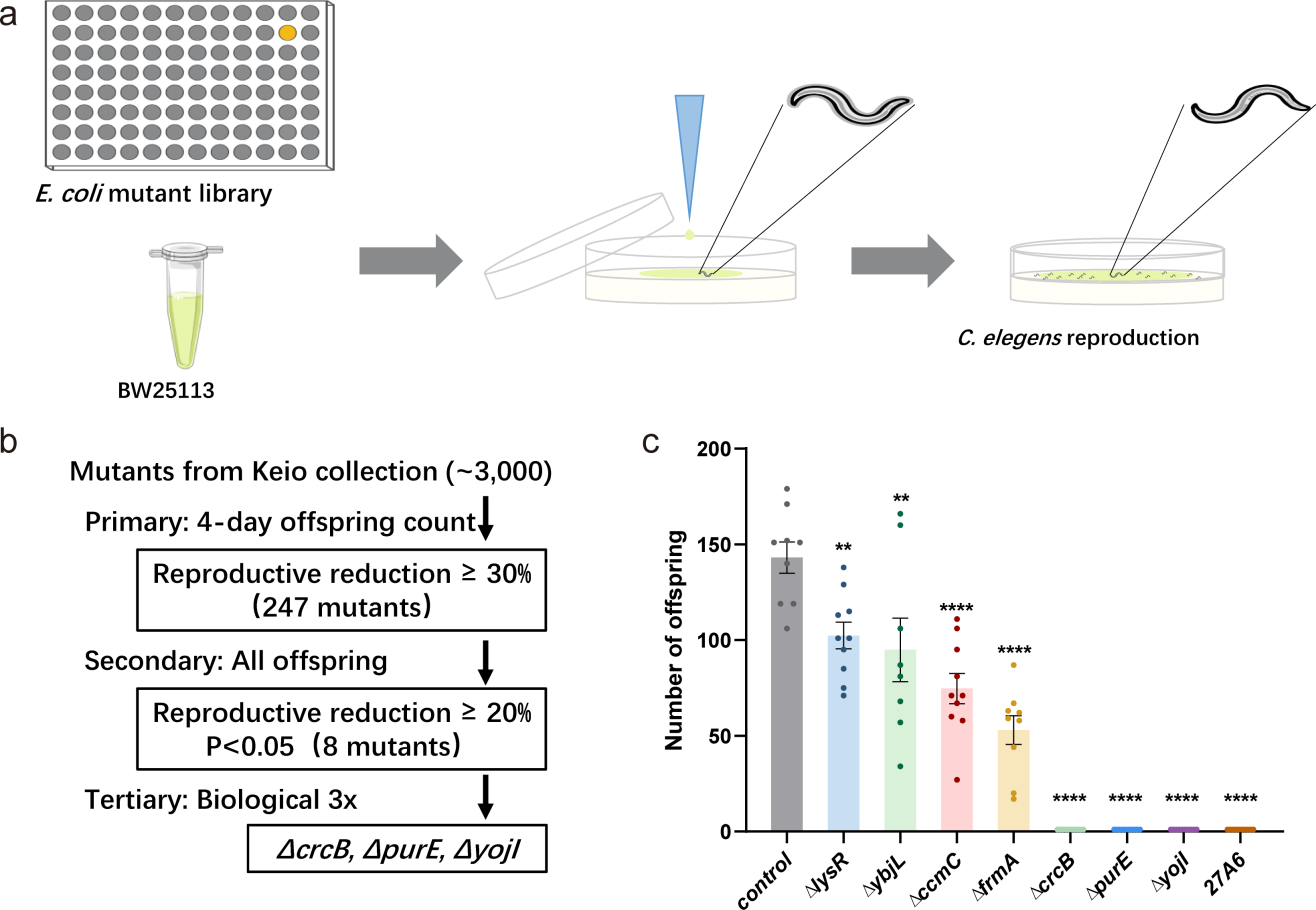
Identification of *E. coli* mutants that delay *C. elegans* fertility. (**a**) Schematic diagram of the high-throughput screening concept, in which *C. elegans* were fed with *E. coli* mutants from the Keio collection. (**b**) Detailed screening workflow corresponding to the schematic in panel **a**. Three biological replicates were performed, and three mutants (*ΔcrcB*, *ΔpurE*, and *ΔyojI*) were finally identified. The entire process reflects the stepwise screening to determine mutants associated with a specific phenotype (reduced reproductive fitness). (**c**) Number of progeny produced by *C. elegans* when fed with eight selected *E. coli* mutants of interest. Statistical significance was determined using Dunnett’s test compared to the BW25113 control group. *P < 0.05, **P < 0.01, ***P < 0.001, and ****P < 0.0001.

To precisely identify microbial single genes that regulate host reproduction, this study used the Keio collection as the core tool to establish a “single bacterium–*C*. *elegans*” one-to-one co-culture system. The parental strain of the collection, *E. coli* K-12 BW25113, served as the control, while each single-gene knockout strain constructed with this strain as the background was used as the experimental group. In the preliminary systematic screening, we performed phenotypic analysis on 3,467 *E. coli* mutants from the Keio collection and initially identified 247 candidate mutants that could significantly inhibit the reproductive fitness of *C. elegans* under co-culture conditions (Fig. 1a).

To further verify the stability of the effect and eliminate false positives, a secondary verification was conducted using the wild-type BW25113 with a consistent genetic background as the control. The criterion was set as “the number of progeny is reduced by ≥20% compared with the control and *P* < 0.05” (Fig. 1b). Finally, eight mutants were confirmed to exhibit a stable reproductive inhibitory effect (Fig. 1c). Among them, *C. elegans* fed with *ΔcrcB*, *ΔpurE*, *ΔyojI*, or 27A6 showed complete inhibition of progeny production. *CrcB* is implicated in cellular stress responses and membrane homeostasis under extreme conditions (19). *PurE* encodes a key phosphoribosylaminoimidazole carboxylase essential for *de novo* purine biosynthesis (20, 21). *YojI* encodes an ABC transporter likely involved in transmembrane transport (22). The mutant strain 27A6 also completely inhibited progeny production in the primary screen but lacks an annotated gene and functional characterization. To ensure reliability in subsequent mechanistic studies, only *ΔcrcB*, *ΔpurE*, and *ΔyojI* were selected for further analysis.

### *ΔcrcB*, *ΔpurE*, and *ΔyojI* mutants delay the onset of *C. elegans* reproduction and reduce progeny number

**Fig 2:**
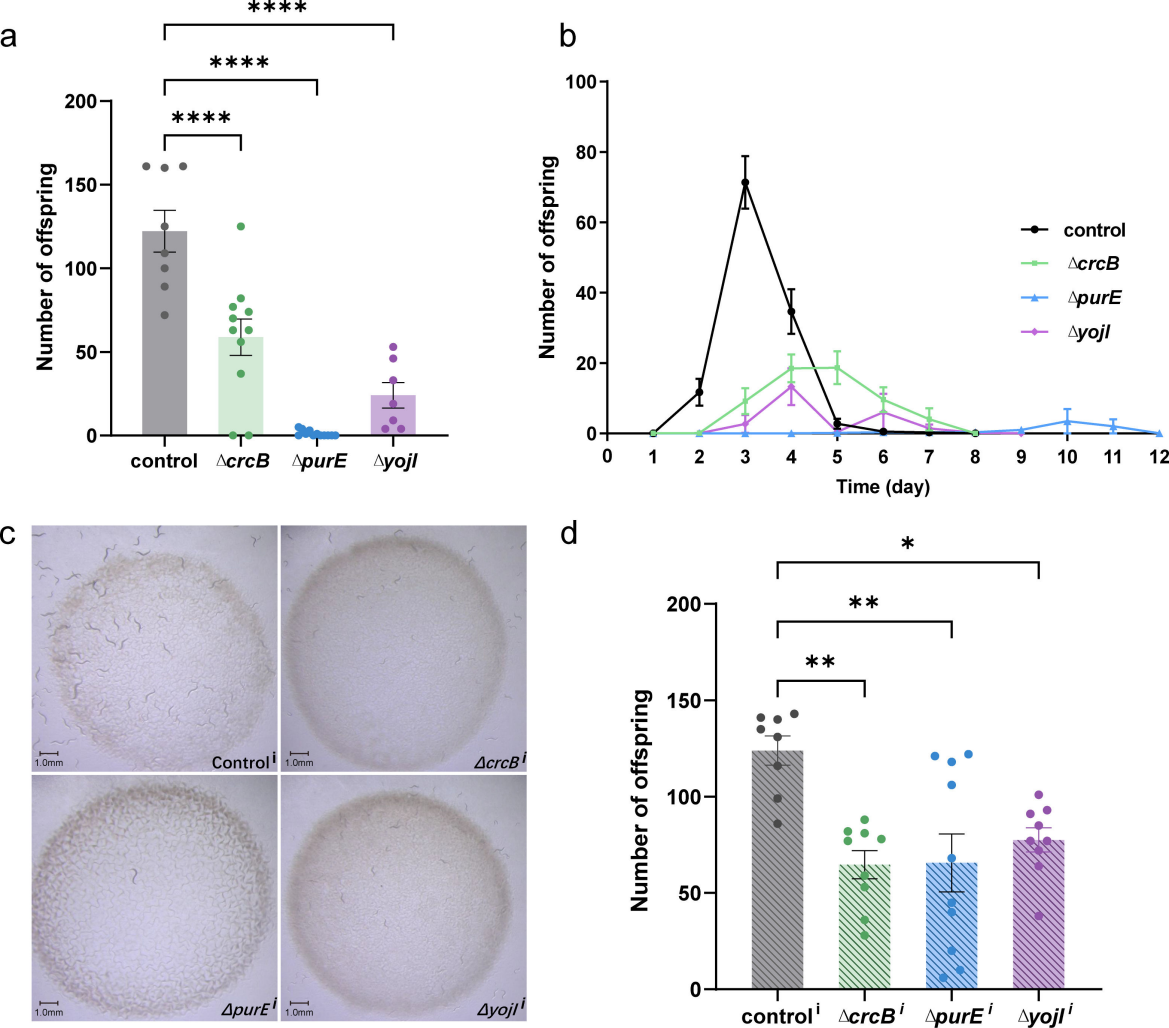
*E. coli* mutants inhibited *C. elegans* reproduction both before and after inactivation. (**a and b**) Live bacterial experiments. When fed with live bacteria, *E. coli* gene mutations significantly inhibited the progeny number of *C. elegans*. (**a**) Gene mutations in *E. coli* may significantly inhibit the progeny number of *C. elegans*. Comparison of progeny numbers between the control group and three mutant groups (*ΔcrcB*, *ΔpurE*, and *ΔyojI*). ****P < 0.0001. (**b**) Changes in progeny number (reproductive cycle) of *C. elegans* fed with BW25113 and *E. coli* mutant strains (*ΔcrcB*, *ΔpurE*, and *ΔyojI*) over a 12-day period. (**c and d**) Heat-inactivated bacterial experiments. To investigate whether this effect depends on live bacterial metabolism, bacteria were heat-inactivated at 65°C for 30 min prior to feeding assays (inactivated groups are denoted with a superscript “*i*”). (**c**) Representative images of progeny produced by individual *C. elegans* on the first day when fed with inactivated *E. coli (ΔcrcB^i^*, *ΔpurE^i^*, and *ΔyojI^i^) and BW25113* (control^i^). (**d**) Progeny number of *C. elegans* on the first day when fed with inactivated (*ΔcrcB^i^*, *ΔpurE^i^*, and *ΔyojI^i^) and BW25113* (control^i^). Statistical significance was determined using Dunnett’s test compared to the BW25113 control group. *P < 0.05 and **P < 0.01.

To clarify the effect of single-gene deletion in *E. coli* on the complete reproductive cycle of *C. elegans*, we first conducted three independent replicate experiments. Statistical results showed that compared with the control group fed with wild-type *E. coli* BW25113, *C. elegans* fed with *ΔcrcB*, *ΔpurE*, and *ΔyojI* mutant strains exhibited a significant reduction in total progeny production (Fig. 2a). This suggests that the deletion of these genes impairs the reproductive fitness of *C. elegans* through specific mechanisms. Based on this finding, this study further performed temporal reproductive pattern analysis: the daily egg-laying amount of *C. elegans* was tracked throughout the entire reproductive cycle until reproduction completely ceased. The results indicated that the onset and termination of reproduction in the control group were earlier than those in the mutant-fed groups (Fig. 2b), demonstrating that the mutant strains not only inhibit the reproductive fitness of *C. elegans* but also delay its reproductive process.

To explore the mode of action of the reproductive inhibitory effect of the mutant strains, the *ΔcrcB*, *ΔpurE*, *ΔyojI* mutants, and wild-type *E. coli* were subjected to pasteurization (65°C water bath for 30 min) before being fed to *C. elegans*. The results showed that compared with the inactivated wild-type group, the inactivated mutant groups still significantly reduced the host’s reproductive fitness (Fig. 2c and d), and the inhibitory effect showed a consistent trend with that of the non-inactivated mutants. This indicates that the reproductive inhibitory effect of the mutant strains does not depend on the continuous metabolic activity of viable bacteria but is mediated by heat-stable components produced by the mutants.

### Mutant strains specifically inhibit spermatogenesis in *C. elegans*, extend its lifespan, and do not affect early locomotor ability

**Fig 3:**
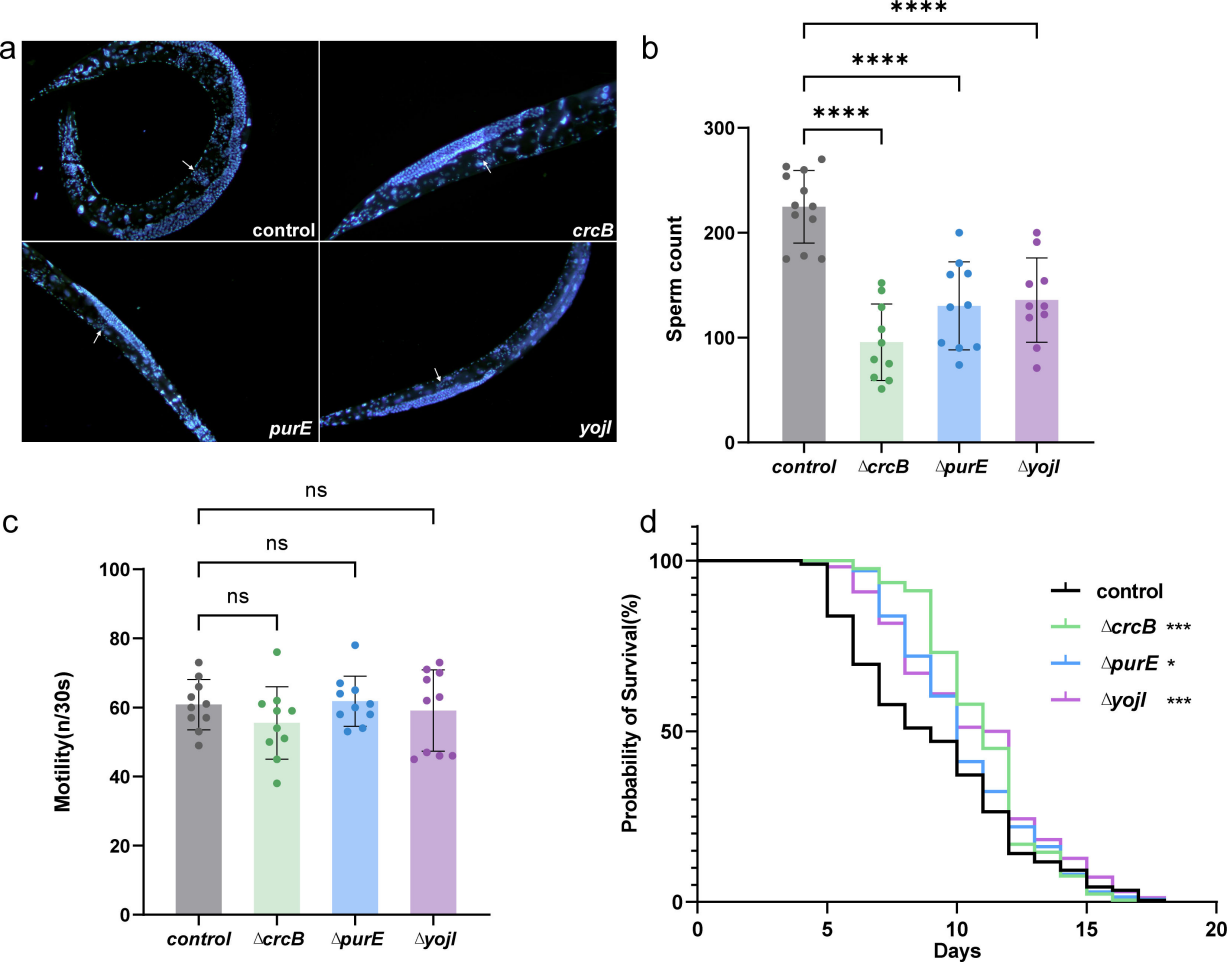
Effects of *E. coli* mutants on sperm characteristics and survival of *C. elegans*. (**a**) DAPI staining of *C. elegans* fed with BW25113 and three mutant strains. The blue-fluorescent particles indicated by white arrows are sperm. (**b**) Counting of *C. elegans* sperm under a fluorescence microscope using ImageJ. ****P < 0.0001. (**c**) Comparison of motility data (vertical axis represents the number of head swings) between the *ΔcrcB*, *ΔpurE*, and *ΔyojI* groups and the control group. The unit is the movement value per 30 s. “ns” indicates no statistically significant effect. (**d**) Survival curves of *C. elegans* fed with *ΔcrcB*, *ΔpurE*, *ΔyojI,* and the control group fed with BW25113 (BW25113, *n* = 204; *ΔcrcB*, *n* = 171; *ΔpurE*, *n* = 136; *ΔyojI*, *n* = 164).

To further dissect the mechanism by which mutant strains inhibit the reproductive fitness of *C. elegans*, this study focused on analyzing their impact on a core step of male germ cell development (the spermatogenesis process). By performing DAPI staining and quantitative analysis when the number of sperm in the *C. elegans* spermatheca reached its peak (Fig. 3a), we found that compared with the control group fed wild-type *E. coli* BW25113, *C. elegans* fed *ΔcrcB*, *ΔpurE*, and *ΔyojI* mutant strains all exhibited a significant reduction in the number of mature sperm in their spermathecae (Fig. 3b). This finding not only confirms the inhibitory effect of mutant strains on *C. elegans* reproductive fitness at the germ cell level but also precisely localizes the phenotype to spermatogenesis—a key developmental step—suggesting that microbial genetic variations may interfere with specific cellular events during spermatogenesis, leading to insufficient production of functional gametes.

To further explore the systemic impact of mutant strains on the overall health status of *C. elegans*, this study simultaneously evaluated two key health indicators: locomotor ability and lifespan. The results showed that at the early stage of feeding, there was no significant difference in locomotor ability among *C. elegans* in each group (Fig. 3c), indicating that the mutant strains did not cause extensive neuromuscular dysfunction. Notably, the lifespan of *C. elegans* in the mutant-treated groups was significantly longer than that in the control group (Fig. 3d). This phenotypic combination of reduced reproductive fitness and extended lifespan, coupled with the characteristic of unaffected early locomotor ability, is highly consistent with the classic “reproduction–lifespan trade-off” theory. The result suggests that microbial gene deletion may induce *C. elegans* to shift resources from reproductive investment to somatic maintenance, thereby ensuring basic life activities while extending lifespan at the cost of reduced fecundity.

### Proteomics reveals *E. coli* mutants regulate *C. elegans* reproduction through energy metabolism

**Fig 4:**
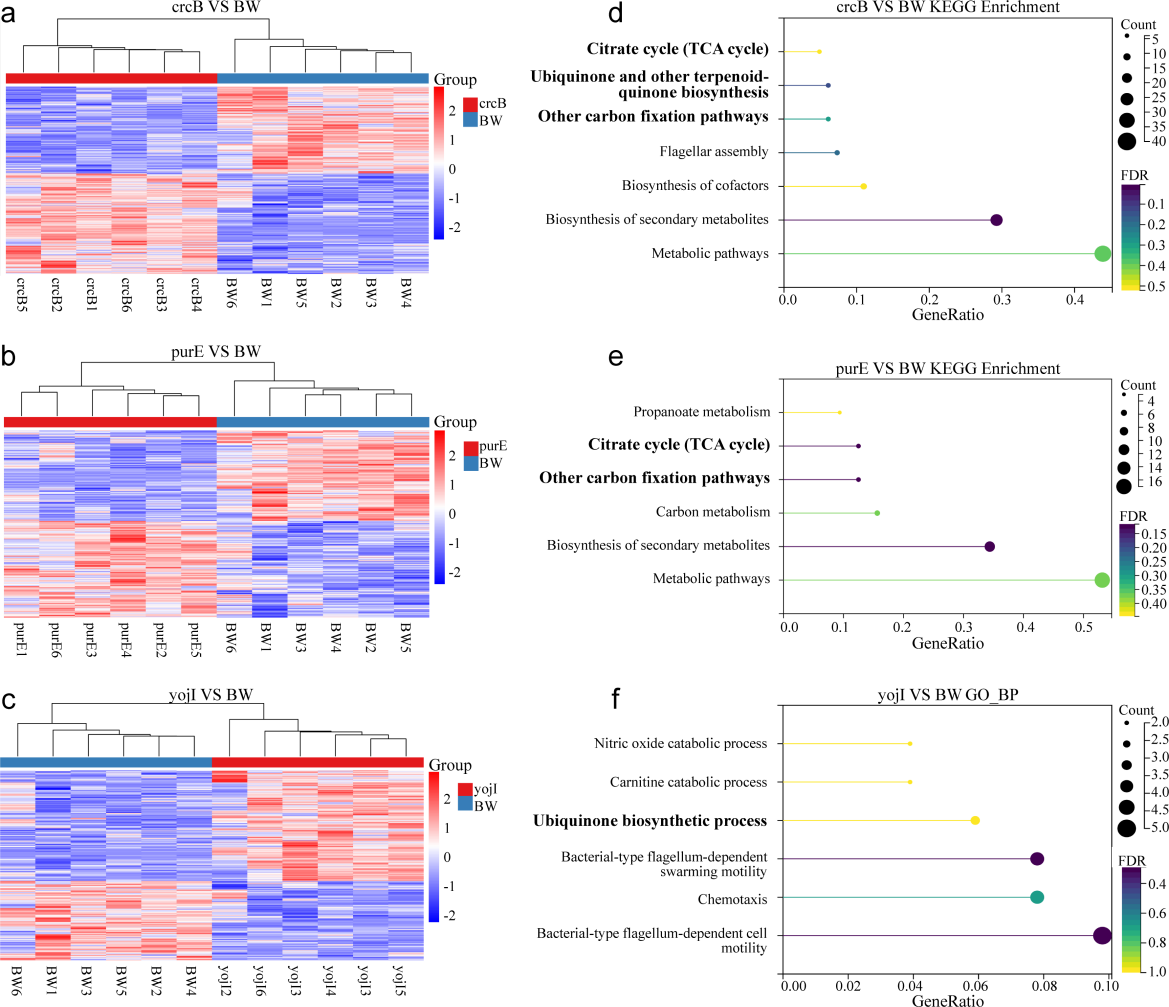
Proteomic analysis of *E. coli* mutant strains compared to wild type. (**a–c**) Heatmaps respectively displaying DEPs between BW25113 and (**a**) *ΔcrcB*, (**b**) *ΔpurE*, and (**c**) *ΔyojI*. (**d and e**) KEGG pathway enrichment analysis of DEPs from (**d**) *ΔcrcB* and (**e**) *ΔpurE*. (**f**) GO-BP enrichment analysis of DEPs from the *ΔyojI*. DEPs were identified using the thresholds |FC| ≥ 1 and *P* < 0.05.

To investigate the molecular pathways by which *E. coli* regulates *C. elegans* reproduction, this study conducted proteomics on the wild-type *E. coli* strain BW25113 and the mutant strains *ΔcrcB*, *ΔpurE*, *ΔyojI*. Compared with the control (BW), the *crcB*, *purE*, and *yojI* groups showed significant differences in differentially expressed proteins (|FC| ≥ 1, *P* < 0.05; Fig. 4a through c), among which 40 were commonly upregulated, and 13 were commonly downregulated across the three groups (Fig. S1a). Enrichment analysis of DEPs showed that *ΔcrcB* and *ΔpurE* were significantly enriched in the KEGG pathways Citrate cycle (TCA cycle) and Other carbon fixation pathways; in addition, *ΔcrcB* was specifically enriched in Ubiquinone and other terpenoid-quinone biosynthesis (Fig. 4d and e). In the *ΔyojI* mutant, since no significant enrichment of these DEPs was detected in KEGG pathways, we instead performed GO-Biological Process (GO-BP) analysis, which revealed significant enrichment of the ubiquinone biosynthetic process (Fig. 4f). These pathways collectively form the core network of energy metabolism. Other carbon fixation pathways provide oxaloacetate and acetyl-CoA as precursors for the TCA cycle (23–25); the TCA cycle oxidizes carbon sources to generate reducing power for oxidative phosphorylation (26); and ubiquinone is also an indispensable component of the electron transport chain (27). The coordinated alterations in these pathways strongly indicate that all three mutations trigger significant changes in bacterial energy metabolism.

### Deletion of *ΔcrcB*, *ΔpurE*, and *ΔyojI* remodels the metabolome of *E. coli*

**Fig 5:**
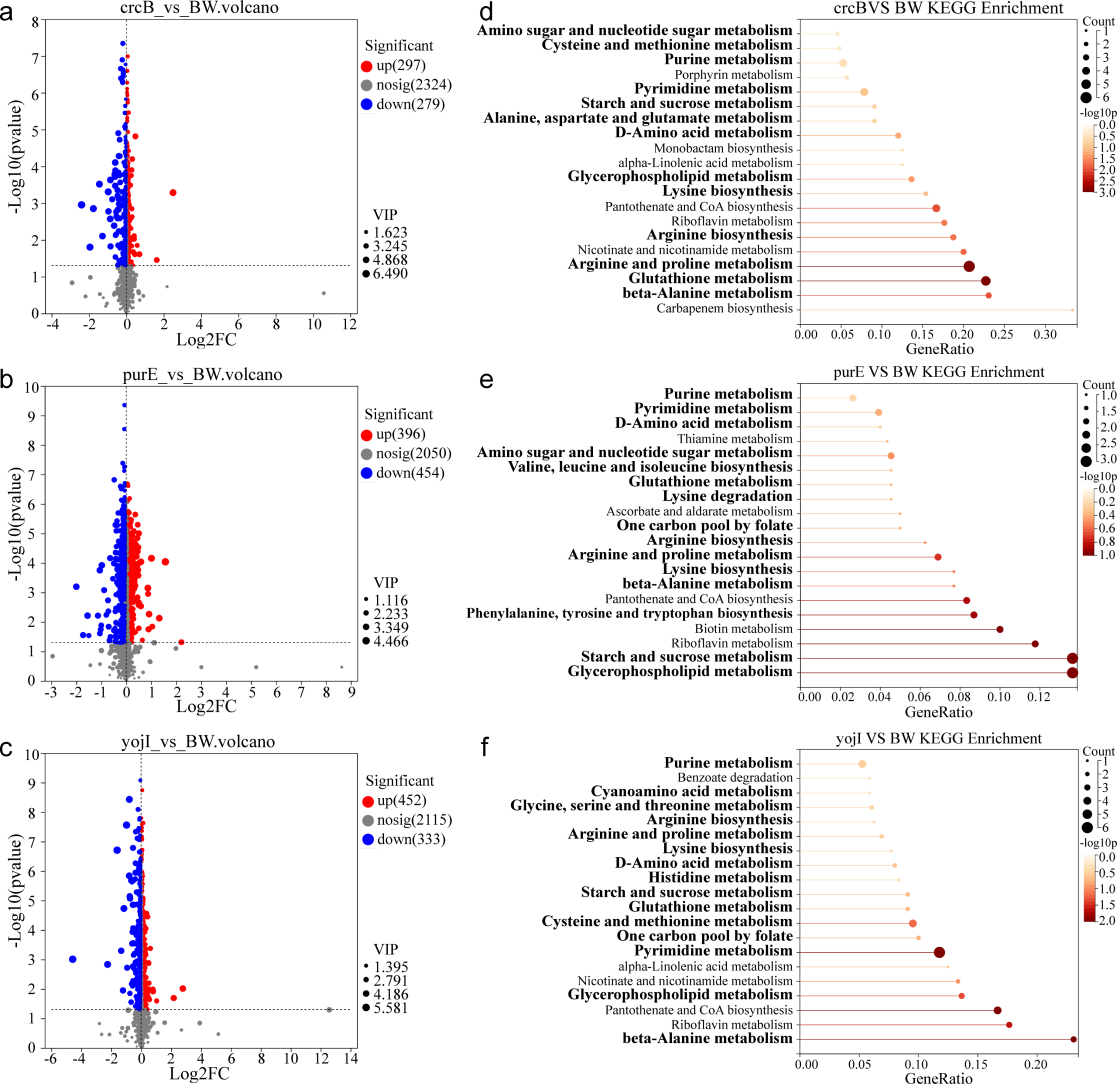
Metabolomic comparison and KEGG pathway enrichment analysis between *E. coli* mutant strains and wild-type BW25113. (**a–c**) Volcano plots of differential metabolites between *ΔcrcB*, *ΔpurE*, *ΔyojI* mutants, and wild-type BW25113 (BW for short), respectively. (**d–f**) Bubble plots of KEGG pathway enrichment analysis for differential metabolites in the corresponding comparison groups, where the size of bubbles represents the number of differential metabolites enriched in each pathway.

Proteomics has pinpointed changes in enzyme levels of mutant strains along the “TCA–carbon fixation–ubiquinone” energy axis; however, whether changes in protein abundance truly translate into metabolic flux rearrangement requires verification at the metabolite level. Therefore, we performed untargeted LC-MS/MS metabolomic analysis on bacterial cells from the same batch to elucidate the characteristics of terminal metabolites following energy network imbalance. Differential metabolite analysis showed that compared with the control group (BW), the experimental groups (*crcB*, *purE*, and *yojI*) all exhibited significant changes in differential metabolites (*P* < 0.05, Vip > 1; Fig. 5a through c). KEGG pathway enrichment analysis was conducted on these differential metabolites, revealing that pathways such as nucleotide metabolism (including Pantothenate and CoA biosynthesis), amino acid metabolism, and glycerophospholipid metabolism were significantly enriched in all three mutant strains (Fig. 5d through f). The result is consistent with the protein group of high-throughput synthetic prediction.

Further focusing on specific metabolites (|log₂FC| > 0.5), CDP-DG was identified to be significantly accumulated in all three mutant strains (Fig. S1b; Table S1). In-depth integration of proteomic and metabolomic data revealed that increased abundance of *eno*, *rpiA*, *atpA*, and *groS* expands the two substrate pools of phosphatidic acid (PA) and CTP, providing sufficient precursors for CDP-DG synthesis (28–31); meanwhile, transcriptional regulation by *sspA* redirects carbon flux from gluconeogenesis to storage lipids, further shunting PA toward CDP-DG and ultimately resulting in the significant upregulation of CDP-DG (32–34). Furthermore, the drawn protein–protein interaction network also confirmed the significance of these proteins (Fig. S1c). Excess bacterial CDP-DG, a precursor of phosphatidylinositol and cardiolipin (CL) (35), is ingested by nematodes, accumulates in the inner mitochondrial membrane, and promotes CL biosynthesis. This enhances the assembly efficiency of Complexes I/III/IV supercomplexes, thereby upregulating oxidative phosphorylation (36). It also affects spermatogenesis by regulating processes such as cell structure and signal transduction (37, 38), suggesting that CDP-DG may serve as a potential molecular mediator in microbe–host reproductive interactions.

### Mutant strains drive upregulation of the host oxidative phosphorylation pathway and downregulation of the cell cycle pathway

To systematically dissect the transcriptional regulatory mechanisms by which microbial genetic variations affect host reproduction, this study performed whole-transcriptome sequencing analysis on *C. elegans* fed with *ΔcrcB*, *ΔpurE*, and *ΔyojI* mutant strains. Through differential expression analysis, KEGG enrichment analysis, and GSEA, common and specific transcriptional reprogramming events in the host in response to different bacterial mutations were revealed.

**Fig 6:**
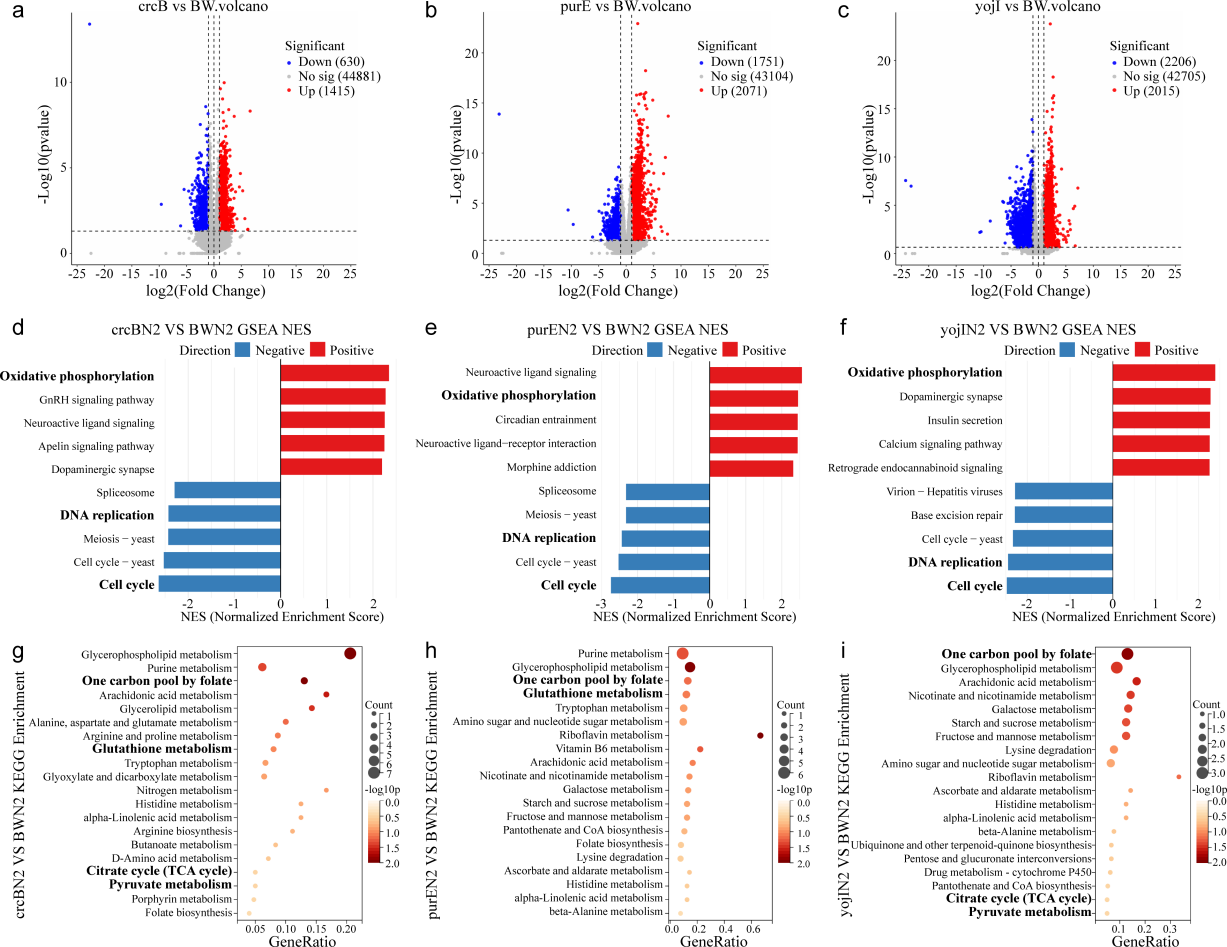
Differential gene and metabolite enrichment analysis of *C. elegans* fed with different mutant bacteria (*crcB*N2/*purE*N2/*yojI*N2) vs. control group (BWN2). (**a**) Volcano plot of differential genes between *crcB*N2 and BWN2. (**b**) Volcano plot of differential genes between *purE*N2 and BWN2. (**c**) Volcano plot of differential genes between *yojI*N2 and BWN2. (**d**) Gene set enrichment analysis (GSEA) plot of *crcB*N2 vs. BWN2. (**e**) GSEA plot of *purE*N2 vs. BWN2. (**f**) GSEA plot of *yojI*N2 vs. BWN2. (**g**) KEGG set enrichment analysis plot of differential metabolites between *crcB*N2 and BWN2. (**h**) KEGG set enrichment analysis plot of differential metabolites between *purE*N2 and BWN2. (**i**) KEGG set enrichment analysis plot of differential metabolites between *yojI*N2 and BWN2.

Results of differential expression analysis showed that compared with the control group fed with wild-type BW25113, each experimental group had a large number of significantly differentially expressed genes (Fig. 6a through c). Further analysis revealed that among these differentially expressed genes, a subset of genes associated with reproductive function showed a significant downregulation trend (Table S2). KEGG pathway enrichment analysis revealed that all three types of mutant bacteria commonly induced significant enrichment of pathways such as Ubiquitin-mediated proteolysis and ATP-dependent chromatin remodeling, which are closely related to cellular energy metabolism and homeostasis regulation. This result further validates that CDP-DG regulates reproductive capacity by regulating energy changes. In addition, different mutant strains also triggered specific pathway responses: the TGF-beta signaling pathway was significantly enriched in the *ΔcrcB* group; the *ΔpurE* group specifically affected the DNA replication and mismatch repair pathways; the *ΔyojI* group upregulated the metabolism of xenobiotics by cytochrome P450 and longevity regulating pathway (Fig. S2a through c).

GSEA further revealed consistent expression change patterns at the global level (Table S3). Under the treatment conditions of the three mutant strains, gene sets related to oxidative phosphorylation in *C. elegans* were significantly upregulated (*P* < 0.05, FDR *Q* < 0.05), while gene sets related to cell cycle and DNA replication showed systematic downregulation (Fig. 6d through f; Fig. S3). This overall transcriptional signature of “high metabolism and low proliferation” indicates that the host undergoes systematic reprogramming at the gene expression level in response to different microbial signals, with energy allocation tending to somatic homeostasis maintenance. This may strengthen the body’s homeostasis guarantee at the cost of sacrificing germ cell proliferation. A correlation network was constructed using genes enriched in the oxidative phosphorylation pathway and significantly differential metabolites in the metabolome, which clearly showed a significant correlation between the two (Fig. S2d through f).

### *E. coli* mutants induce *C. elegans* to enter a “high metabolism, low proliferation” metabolic state

To verify the “high metabolism, low proliferation” state inferred from transcriptome data at the host level, this study performed metabolomics analysis on *C. elegans* fed with *ΔcrcB*, *ΔpurE*, and *ΔyojI* mutant strains. The results showed that the energy metabolism-related pathways of *C. elegans* in all three experimental groups underwent significant remodeling. Among them, core energy-producing pathways such as the citrate cycle (TCA cycle), glutathione metabolism, and pyruvate metabolism were significantly enriched (Fig. 6g through i), indicating an upregulation of the host’s overall energy metabolism level. At the same time, the one-carbon pool by the folate pathway—a key source of nucleotide synthesis and methyl donors—also exhibited metabolic abnormalities (39). Dysfunction of this pathway may directly affect the supply of nucleotide precursors, thereby limiting the raw materials required for DNA replication and cell division. The characteristic of the host metabolome, in which energy metabolism pathways are upregulated while nucleotide synthesis pathways are disturbed, directly confirms at the metabolite level that *C. elegans* is in a functional state of “high metabolism and low proliferation.” This provides key metabolic evidence for explaining the decreased reproductive fitness of *C. elegans*.

## DISCUSSION

Through systematic functional screening, this study identified a one-to-one correspondence between single-gene variations in microbes and the reproductive fitness of *C. elegans*. Using the powerful tool of the Keio collection, we identified that the deletion of three key genes (*crcB*, *purE*, and *yojI*) significantly inhibits the reproductive fitness of *C. elegans* in an ideal model that excludes the interference of complex microbial communities. This finding advances the research on the impact of microbes on host reproduction from the community ecological level to the single-gene functional level. Integrative multi-omics analysis also suggests a regulatory pathway in which microbial gene deletion induces remodeling of the *E. coli* metabolome, which in turn leads to reprogramming of the host transcriptional program and ultimately causes changes in reproductive phenotypes. This result provides a new paradigm for understanding microbe–host interactions and emphasizes that the microbial genetic background itself is a key variable affecting host health.

Traditional studies have mostly focused on the overall effects of intact microbial communities or specific bacterial species, making it difficult to dissect the functional contribution of individual genes (40–42). In this study, the Keio collection—covering thousands of pre-defined gene mutations—was used, which is equivalent to accelerating the simulation of the natural evolution process of microbes under laboratory conditions. The *ΔcrcB*, *ΔpurE*, and *ΔyojI* mutants screened from it all exhibited significant reproductive inhibitory effects, and this effect persisted even after bacterial inactivation. This indicates that their action depends on heat-stable components produced by *E. coli* (such as accumulated metabolites or cell wall components), rather than the continuous metabolic activity of viable bacteria. This result suggests that specific microbial metabolic states formed by gene mutations in nature may exert profound and long-lasting impacts on the reproductive health of offspring through food chains or symbiotic relationships.

Although the original gene functions of the three mutant strains differ (involved in stress response, purine synthesis, and unknown function, respectively) (19, 43), the omics data strongly suggest that these three mutants may induce a “high energy consumption and low proliferation” state in the host by disrupting host oxidative phosphorylation as well as cell cycle and DNA replication. We hypothesize that metabolic stress caused by bacterial gene deletion (possibly due to insufficient nutrient supply or accumulation of harmful metabolites) forces the host to enhance energy production to maintain basic homeostasis. However, this compensatory response is costly, leading to the redistribution of energy and raw materials originally used for the rapid division of germ cells, thereby resulting in decreased reproductive fitness. This mechanism is highly consistent with the “reproduction–lifespan trade-off” theory: when organisms perceive environmental stress, they prioritize somatic maintenance and stress defense, sacrificing reproductive investment (44–46). This also reasonably explains the phenomenon of extended *C. elegans* lifespan observed in this study. However, the current evidence remains correlative and requires further research for validation. For example, *C. elegans* could be treated with specific inhibitors of oxidative phosphorylation, such as rotenone (47), to test whether they can suppress the mutant-induced reproductive phenotype. Alternatively, RNAi-mediated knockdown of key cell cycle genes could be employed to investigate their direct relationship with the observed reduction in fecundity (48).

Within the aforementioned unified regulatory framework, different mutant strains also finely regulate host physiological responses through specific molecular pathways, reflecting the precision of microbial genetic variation regulation. The *ΔcrcB* mutation specifically enriches the TGF-β signaling pathway, which plays a core regulatory role in *C. elegans* development, immunity, and reproduction (49–51). Deletion of *crcB* may lead to changes in bacterial membrane structure or enhanced stress status, and the signaling molecules released by the mutant may interfere with the host’s TGF-β signaling network, thereby specifically affecting reproductive function. The *ΔpurE* mutation exhibits the strongest inhibitory effect on reproduction. *PurE* is a key enzyme in *de novo* purine synthesis, and its deletion deprives bacteria of the ability to supply adequate nucleotide precursors to the host, thereby disrupting host nucleotide metabolism and downregulating DNA replication and mismatch repair (43, 52). Given that the reproductive system is the tissue with the most active cell division in the body and is extremely sensitive to nucleotide supply, it is thus most severely affected. Conversely, the *ΔyojI* mutation may result in the accumulation of specific metabolites within bacteria, which imposes survival stress on the organisms and thereby elicits the host’s activation of detoxification and stress defense systems, including metabolism of xenobiotic by cytochrome P450 and longevity-related pathways (53, 54). Such global protective responses also achieve energy redistribution at the cost of sacrificing reproductive resources.

This study is based on a simplified model system in which *C. elegans* was fed a single bacterial strain (*E. coli*). While this approach facilitates the precise dissection of causal relationships between individual microbial genes and host phenotypes under controlled conditions, it cannot fully recapitulate the complexity of a multi-kingdom gut microbial community in nature. In a real ecosystem, interactions such as metabolic cross-feeding and competitive exclusion among microbes may potentiate or mask the effects observed with a single mutant strain (55, 56). Furthermore, the proposed link between bacterial CDP-DG accumulation and the upregulation of host oxidative phosphorylation is currently supported primarily by correlative evidence from multi-omics integration. Direct functional validation, such as exogenous CDP-DG supplementation to *C. elegans* fed control bacteria or genetic manipulation of bacterial CDP-DG synthesis pathways, is required to establish causality.

When considering the translation of these findings to higher organisms such as mammals, this study offers a valuable theoretical framework while also facing significant challenges. On one hand, the core metabolic pathways implicated here, including oxidative phosphorylation, purine metabolism, and glycerophospholipid metabolism, are evolutionarily conserved (57–59). This suggests that dysregulation of homologous genes in the human gut microbiota could potentially affect host reproductive health through similar mechanisms, providing a proof of principle for understanding the molecular basis of reproductive disorders such as polycystic ovary syndrome. On the other hand, mammals possess far more complex neuro-endocrine-immune networks than *C. elegans*, through which the gut microbiota may exert indirect effects on reproduction (60, 61). The direct metabolite-host interaction model observed in this study may therefore be modulated or overridden by other regulatory signals in higher organisms.

Despite these limitations, this study identifies potential targets for reproductive health intervention: CDP-DG accumulation may serve as a biomarker for microbial-mediated host metabolic stress; the imbalance between oxidative phosphorylation and cell cycle gene expression suggests the value of modulating host energy metabolism (62); and most importantly, this work opens a new avenue for intervention along the microbe–host metabolic axis. Future strategies could indirectly protect host reproductive function by reshaping microbial metabolism, for example, by reducing CDP-DG production.

## Supplementary Material

Reviewer comments

## Data Availability

Transcriptomic and metabolomics data of *C. elegans* and metabolomics data of *E. coli* are provided in Tables S4 to S6.

## SUPPLEMENTAL MATERIAL

Figure S1 msystems.01698-25-s0001.pdf Differential proteins and metabolites and an interaction network of shared upregulated proteins.

Figure S2 msystems.01698-25-s0002.pdf Differential gene KEGG pathway and gene-metabolite correlation analysis.

Figure S3 msystems.01698-25-s0003.pdf GSEA analysis of oxidative phosphorylation and cell cycle.

Supplemental Legends msystems.01698-25-s0004.docx Legends for Figures S1-S3 and Tables S1-S6.

Table S1 msystems.01698-25-s0005.xlsx Differential metabolites in three mutant *E. coli* strains compared to wild type.

Table S2 msystems.01698-25-s0006.xlsx Downregulated reproduction-related genes in *C. elegans* fed mutant bacteria.

Table S3 msystems.01698-25-s0007.xlsx Significantly altered pathways and modules identified by GSEA.

Table S4 msystems.01698-25-s0008.xlsx Multi-omics data of *C. elegans* and Δ*crcB*.

Table S5 msystems.01698-25-s0009.xlsx Multi-omics data of *C. elegans* and Δ*purE*.

Table S6 msystems.01698-25-s0010.xlsx Multi-omics data of *C. elegans* and Δ*yojI*.

OPEN PEER REVIEW reviewer-comments.pdf An accounting of the reviewer comments and feedback.

ASM does not own the copyrights to Supplemental Material that may be linked to, or accessed through, an article. The authors have granted ASM a non-exclusive, world-wide license to publish the Supplemental Material files. Please contact the corresponding author directly for reuse.

## References

[1] Manrique P, Montero I, Fernandez-Gosende M, Martinez N, Cantabrana CH, Rios-Covian D. 2024. Past, present, and future of microbiome-based therapies. Microbiome Res Rep 3:23. doi:10.20517/mrr.2023.80 38841413 PMC11149097

[2] Sonowal R, Swimm AI, Cingolani F, Parulekar N, Cleverley TL, Sahoo A, Ranawade A, Chaudhuri D, Mocarski ES, Koehler H, Nitsche K, Mesiano S, Kalman D. 2023. A microbiota and dietary metabolite integrates DNA repair and cell death to regulate embryo viability and aneuploidy during aging. Sci Adv 9:eade8653. doi:10.1126/sciadv.ade8653 36827370 PMC9956122

[3] Bai R, Wang T, Gu R, Cai Y, Chen J, Cai W, Zhou D, Li Y, Luo J, Wang X, Zhu Z. 2025. Microbial genetic composition regulates host social behavior. Gut Microbes 17:2536091. doi:10.1080/19490976.2025.2536091 40702822 PMC12309553

[4] Li Y, Bai R, Zhu Y, Shi P, Wang T, Zhou D, Zhou J, Zhu T, Zhang X, Gu R, Ding X, Chen H, Wang X, Zhu Z. 2025. Genetic variation in gut microbe as a key regulator of host social behavior in C. elegans Gut Microbes 17:2490828. doi:10.1080/19490976.2025.2490828 40223740 PMC12005443

[5] Braun NJ, Mor DE. 2025. Gut microbiome species Levilactobacillus brevis regulates reproductive fitness in C. elegans. Curr Res Microb Sci 9:100471. doi:10.1016/j.crmicr.2025.100471 41018064 PMC12464547

[6] Khanna A, Kumar J, Vargas MA, Barrett L, Katewa S, Li P, McCloskey T, Sharma A, Naudé N, Nelson C, Brem R, Killilea DW, Mooney SD, Gill M, Kapahi P. 2016. A genome-wide screen of bacterial mutants that enhance dauer formation in C. elegans. Sci Rep 6:38764. doi:10.1038/srep38764 27958277 PMC5153853

[7] Ohno H, Bao Z. 2022. Small RNAs couple embryonic developmental programs to gut microbes. Sci Adv 8:eabl7663. doi:10.1126/sciadv.abl7663 35319987 PMC8942359

[8] Venzon M, Das R, Luciano DJ, Burnett J, Park HS, Devlin JC, Kool ET, Belasco JG, Hubbard EJA, Cadwell K. 2022. Microbial byproducts determine reproductive fitness of free-living and parasitic nematodes. Cell Host Microbe 30:786–797. doi:10.1016/j.chom.2022.03.015 35413267 PMC9187612

[9] Wang L, Yang Z, Pang Y, Wu X, Zhang X, Zhu T, Ding X, Liu W, Zhou Y, Zhang P, Li Y, Zhu Z. 2025. Gut microbial genetic variation prolongs host healthy longevity and remodels metabolome and proteome in Drosophila melanogaster. Adv Sci (Weinheim) 12:e05469. doi:10.1002/advs.202505469 PMC12713025 41013900

[10] Li Y, Xu S, Wang L, Shi H, Wang H, Fang Z, Hu Y, Jin J, Du Y, Deng M, Wang L, Zhu Z. 2023. Gut microbial genetic variation modulates host lifespan, sleep, and motor performance. ISME J 17:1733–1740. doi:10.1038/s41396-023-01478-x 37550381 PMC10504343

[11] Baba T, Ara T, Hasegawa M, Takai Y, Okumura Y, Baba M, Datsenko KA, Tomita M, Wanner BL, Mori H. 2006. Construction of Escherichia coli K-12 in-frame, single-gene knockout mutants: the Keio collection. Mol Syst Biol 2:2006. doi:10.1038/msb4100050 PMC1681482 16738554

[12] Zhang J, Holdorf AD, Walhout AJ. 2017. C. elegans and its bacterial diet as a model for systems-level understanding of host–microbiota interactions. Curr Opin Biotechnol 46:74–80. doi:10.1016/j.copbio.2017.01.008 28189107 PMC5544573

[13] Jin Z, Yu H, Haul C, Wang L, Zhu Z, Shen Q, Cao X. 2023. WormTrack: dataset and benchmark for multi-object tracking in worm crowds. Proceedings of the 31st ACM International Conference on Multimedia; Ottawa, ON ,Canada: , p 5756–5763, New York, NY, USA. doi:10.1145/3581783.3613812

[14] Kim D, Paggi JM, Park C, Bennett C, Salzberg SL. 2019. Graph-based genome alignment and genotyping with HISAT2 and HISAT-genotype. Nat Biotechnol 37:907–915. doi:10.1038/s41587-019-0201-4 31375807 PMC7605509

[15] Li B, Dewey CN. 2011. RSEM: accurate transcript quantification from RNA-Seq data with or without a reference genome. BMC Bioinform 12:323. doi:10.1186/1471-2105-12-323 PMC3163565 21816040

[16] Love MI, Huber W, Anders S. 2014. Moderated estimation of fold change and dispersion for RNA-seq data with DESeq2. Genome Biol 15:550. doi:10.1186/s13059-014-0550-8 25516281 PMC4302049

[17] Subramanian A, Tamayo P, Mootha VK, Mukherjee S, Ebert BL, Gillette MA, Paulovich A, Pomeroy SL, Golub TR, Lander ES, Mesirov JP. 2005. Gene set enrichment analysis: a knowledge-based approach for interpreting genome-wide expression profiles. Proc Natl Acad Sci USA 102:15545–15550. doi:10.1073/pnas.0506580102 16199517 PMC1239896

[18] Han C, Shi C, Liu L, Han J, Yang Q, Wang Y, Li X, Fu W, Gao H, Huang H, Zhang X, Yu K. 2024. Majorbio cloud 2024: Update single-cell and multiomics workflows. Imeta 3:e217. doi:10.1002/imt2.217 39135689 PMC11316920

[19] Sand O, Gingras M, Beck N, Hall C, Trun N. 2003. Phenotypic characterization of overexpression or deletion of the Escherichia coli crcA, cspE and crcB genes. Microbiology (Reading) 149:2107–2117. doi:10.1099/mic.0.26363-0 12904550

[20] Meyer E, Leonard NJ, Bhat B, Stubbe J, Smith JM. 1992. Purification and characterization of the purE, purK, and purC gene products: identification of a previously unrecognized energy requirement in the purine biosynthetic pathway. Biochemistry 31:5022–5032. doi:10.1021/bi00136a016 1534690

[21] Watanabe W, Sampei G, Aiba A, Mizobuchi K. 1989. Identification and sequence analysis of Escherichia coli purE and purK genes encoding 5’-phosphoribosyl-5-amino-4-imidazole carboxylase for de novo purine biosynthesis. J Bacteriol 171:198–204. doi:10.1128/jb.171.1.198-204.1989 2644189 PMC209573

[22] Delgado MA, Vincent PA, Farías RN, Salomón RA. 2005. YojI of Escherichia coli functions as a microcin J25 efflux pump. J Bacteriol 187:3465–3470. doi:10.1128/JB.187.10.3465-3470.2005 15866933 PMC1112001

[23] Fuchs G. 2011. Alternative pathways of carbon dioxide fixation: insights into the early evolution of life? Annu Rev Microbiol 65:631–658. doi:10.1146/annurev-micro-090110-102801 21740227

[24] Zarzycki J, Brecht V, Müller M, Fuchs G. 2009. Identifying the missing steps of the autotrophic 3-hydroxypropionate CO2 fixation cycle in Chloroflexus aurantiacus. Proc Natl Acad Sci USA 106:21317–21322. doi:10.1073/pnas.0908356106 19955419 PMC2795484

[25] Schulz-Mirbach H, Wichmann P, Satanowski A, Meusel H, Wu T, Nattermann M, Burgener S, Paczia N, Bar-Even A, Erb TJ. 2024. New-to-nature CO2-dependent acetyl-CoA assimilation enabled by an engineered B12-dependent acyl-CoA mutase. Nat Commun 15:10235. doi:10.1038/s41467-024-53762-9 39592584 PMC11599936

[26] Alves TC, Pongratz RL, Zhao X, Yarborough O, Sereda S, Shirihai O, Cline GW, Mason G, Kibbey RG, Integrated S-W. 2015. Mass-isotopomeric flux analysis of the TCA cycle. Cell Metab 22:936–947. doi:10.1016/j.cmet.2015.08.021 26411341 PMC4635072

[27] Verkhovsky M, Bloch DA, Verkhovskaya M. 2012. Tightly-bound ubiquinone in the Escherichia coli respiratory complex I. Biochim Biophys Acta 1817:1550–1556. doi:10.1016/j.bbabio.2012.04.013 22580197

[28] Carman GM, Henry SA. 2007. Phosphatidic acid plays a central role in the transcriptional regulation of glycerophospholipid synthesis in Saccharomyces cerevisiae. J Biol Chem 282:37293–37297. doi:10.1074/jbc.R700038200 17981800 PMC3565216

[29] Chang YF, Carman GM. 2008. CTP synthetase and its role in phospholipid synthesis in the yeast Saccharomyces cerevisiae. Prog Lipid Res 47:333–339. doi:10.1016/j.plipres.2008.03.004 18439916 PMC2583782

[30] Zhang Y, Zhang X, Li Z, Ye Q. 2008. Effects of overexpression of atpA, purHD and ndh on the growth of Escherichia coli DH5alpha. Wei Sheng Wu Xue Bao 48:1042–1047. 18956753

[31] Dahiya V, Chaudhuri TK. 2014. Chaperones GroEL/GroES accelerate the refolding of a multidomain protein through modulating on-pathway intermediates. J Biol Chem 289:286–298. doi:10.1074/jbc.M113.518373 24247249 PMC3879552

[32] Lopez SC, Lee Y, Zhang K, Shipman SL. 2025. SspA is a transcriptional regulator of CRISPR adaptation in E. coli. Nucleic Acids Res 53:gkae1244. doi:10.1093/nar/gkae1244 39727179 PMC11879090

[33] Hansen AM, Qiu Y, Yeh N, Blattner FR, Durfee T, Jin DJ. 2005. SspA is required for acid resistance in stationary phase by downregulation of H-NS in Escherichia coli. Mol Microbiol 56:719–734. doi:10.1111/j.1365-2958.2005.04567.x 15819627

[34] Wahl A, My L, Dumoulin R, Sturgis JN, Bouveret E. 2011. Antagonistic regulation of dgkA and plsB genes of phospholipid synthesis by multiple stress responses in Escherichia coli. Mol Microbiol 80:1260–1275. doi:10.1111/j.1365-2958.2011.07641.x 21463370

[35] Jennings W, Epand RM. 2020. CDP-diacylglycerol, a critical intermediate in lipid metabolism. Chem Phys Lipids 230:104914. doi:10.1016/j.chemphyslip.2020.104914 32360136

[36] Pfeiffer K, Gohil V, Stuart RA, Hunte C, Brandt U, Greenberg ML, Schägger H. 2003. Cardiolipin stabilizes respiratory chain supercomplexes. J Biol Chem 278:52873–52880. doi:10.1074/jbc.M308366200 14561769

[37] Nath AS, Parsons BD, Makdissi S, Chilvers RL, Mu Y, Weaver CM, Euodia I, Fitze KA, Long J, Scur M, Mackenzie DP, Makrigiannis AP, Pichaud N, Boudreau LH, Simmonds AJ, Webber CA, Derfalvi B, Hamon Y, Rachubinski RA, Di Cara F. 2022. Modulation of the cell membrane lipid milieu by peroxisomal β-oxidation induces Rho1 signaling to trigger inflammatory responses. Cell Rep 38:110433. doi:10.1016/j.celrep.2022.110433 35235794

[38] Xu J, Chen S, Wang W, Man Lam S, Xu Y, Zhang S, Pan H, Liang J, Huang X, Wang Y, Li T, Jiang Y, Wang Y, Ding M, Shui G, Yang H, Huang X. 2022. Hepatic CDP-diacylglycerol synthase 2 deficiency causes mitochondrial dysfunction and promotes rapid progression of NASH and fibrosis. Sci Bull Sci Found Philipp 67:299–314. doi:10.1016/j.scib.2021.10.014 36546079

[39] Maynard AG, Pohl NK, Mueller AP, Petrova B, Wong AYL, Wang P, Culhane AJ, Brook JR, Hirsch LM, Hoang N, Kirkland O, Braun T, Ducamp S, Fleming MD, Li H, Kanarek N. 2024. Folate depletion induces erythroid differentiation through perturbation of de novo purine synthesis. Sci Adv 10:eadj9479. doi:10.1126/sciadv.adj9479 38295180 PMC10830111

[40] Chen G, Qi H, Jiang L, Sun S, Zhang J, Yu J, Liu F, Zhang Y, Du S. 2024. Integrating single-cell RNA-Seq and machine learning to dissect tryptophan metabolism in ulcerative colitis. J Transl Med 22:1121. doi:10.1186/s12967-024-05934-w 39707393 PMC11662780

[41] Chen C, Liao J, Xia Y, Liu X, Jones R, Haran J, McCormick B, Sampson TR, Alam A, Ye K. 2022. Gut microbiota regulate Alzheimer’s disease pathologies and cognitive disorders via PUFA-associated neuroinflammation. Gut 71:2233–2252. doi:10.1136/gutjnl-2021-326269 35017199 PMC10720732

[42] Hu M, Xu Y, Wang Y, Huang Z, Wang L, Zeng F, Qiu B, Liu Z, Yuan P, Wan Y, Ge S, Zhong D, Xiao S, Luo R, He J, Sun M, Zhuang X, Guo N, Cui C, Gao J, Zhou H, He X. 2025. Gut microbial-derived N-acetylmuramic acid alleviates colorectal cancer via the AKT1 pathway. Gut 74:1230–1245. doi:10.1136/gutjnl-2024-332891 40015949

[43] Mathews II, Kappock TJ, Stubbe J, Ealick SE. 1999. Crystal structure of Escherichia coli PurE, an unusual mutase in the purine biosynthetic pathway. Structure 7:1395–1406. doi:10.1016/s0969-2126(00)80029-5 10574791

[44] Braendle C, Paaby A. 2024. Life history in Caenorhabditis elegans: from molecular genetics to evolutionary ecology. Genetics 228:iyae151. doi:10.1093/genetics/iyae151 39422376 PMC11538407

[45] Wu D, Wang Z, Huang J, Huang L, Zhang S, Zhao R, Li W, Chen D, Ou G. 2022. An antagonistic pleiotropic gene regulates the reproduction and longevity tradeoff. Proc Natl Acad Sci USA 119:e2120311119. doi:10.1073/pnas.2120311119 35482917 PMC9170148

[46] Sarmah S, Truong HT-HH, McColl G, Burke R, Mirth CK, Piper MDW. 2025. Dietary zinc limitation dictates lifespan and reproduction trade-offs of drosophila mothers. Aging Cell 24:e14498. doi:10.1111/acel.14498 39891318 PMC12073914

[47] Li N, Ragheb K, Lawler G, Sturgis J, Rajwa B, Melendez JA, Robinson JP. 2003. Mitochondrial complex I inhibitor rotenone induces apoptosis through enhancing mitochondrial reactive oxygen species production. J Biol Chem 278:8516–8525. doi:10.1074/jbc.M210432200 12496265

[48] Sanscrainte ND, Arimoto H, Waits CM, Li LY, Johnson D, Geden C, Becnel JJ, Estep AS. 2018. Reduction in Musca domestica fecundity by dsRNA-mediated gene knockdown. PLoS One 13:e0187353. doi:10.1371/journal.pone.0187353 29342168 PMC5771563

[49] Dalfó D, Michaelson D, Hubbard EJA. 2012. Sensory regulation of the C. elegans germline through TGF-β-dependent signaling in the niche. Curr Biol 22:712–719. doi:10.1016/j.cub.2012.02.064 22483938 PMC3633564

[50] Yamamoto KK, Savage-Dunn C. 2023. TGF-β pathways in aging and immunity: lessons from Caenorhabditis elegans. Front Genet 14:1220068. doi:10.3389/fgene.2023.1220068 37732316 PMC10507863

[51] Nonninger TJ, Mak J, Gerisch B, Ramponi V, Kawamura K, Ripa R, Schilling K, Latza C, Kölschbach J, Serrano M, Antebi A. 2025. A TFEB-TGFβ axis systemically regulates diapause, stem cell resilience and protects against a senescence-like state. Nat Aging 5:1340–1357. doi:10.1038/s43587-025-00911-4 40588651 PMC12270908

[52] Wang X, Ji X, Feng S, Sun Y, Zhu L, Liu J. 2025. Immunological and protective evaluation of purE/purK gene-deletion mutant of Brucella melitensis M5 strain. Microb Pathog 200:107308. doi:10.1016/j.micpath.2025.107308 39828225

[53] Esteves F, Rueff J, Kranendonk M. 2021. The central role of cytochrome P450 in xenobiotic metabolism-a brief review on a fascinating enzyme family. J Xenobiot 11:94–114. doi:10.3390/jox11030007 34206277 PMC8293344

[54] Aranaz P, Navarro-Herrera D, Zabala M, Romo-Hualde A, López-Yoldi M, Vizmanos JL, Milagro FI, González-Navarro CJ. 2020. Phenolic compounds reduce the fat content in Caenorhabditis elegans by affecting lipogenesis, lipolysis, and different stress responses. Pharmaceuticals (Basel) 13:11. doi:10.3390/ph13110355 33143060 PMC7693530

[55] Culp EJ, Goodman AL. 2023. Cross-feeding in the gut microbiome: ecology and mechanisms. Cell Host Microbe 31:485–499. doi:10.1016/j.chom.2023.03.016 37054671 PMC10125260

[56] Coyte KZ, Schluter J, Foster KR. 2015. The ecology of the microbiome: Networks, competition, and stability. Science 350:663–666. doi:10.1126/science.aad2602 26542567

[57] Barshad G, Blumberg A, Cohen T, Mishmar D. 2022. Corrigendum: human primitive brain displays negative mitochondrial-nuclear expression correlation of respiratory genes. Genome Res 32:1626. doi:10.1101/gr.277125.122 39074347 PMC9435736

[58] Sendinc E, Shi Y. 2023. RNA m6A methylation across the transcriptome. Mol Cell 83:428–441. doi:10.1016/j.molcel.2023.01.006 36736310

[59] Senoo N, Chinthapalli DK, Baile MG, Golla VK, Saha B, Oluwole AO, Ogunbona OB, Saba JA, Munteanu T, Valdez Y, Whited K, Sheridan MS, Chorev D, Alder NN, May ER, Robinson CV, Claypool SM. 2024. Functional diversity among cardiolipin binding sites on the mitochondrial ADP/ATP carrier. EMBO J 43:2979–3008. doi:10.1038/s44318-024-00132-2 38839991 PMC11251061

[60] Li X, Zhang H, Hodder T, Wang W, Myers CL, Yilmaz LS, Walhout AJM. 2025. Systems-level design principles of metabolic rewiring in an animal. Nature 640:203–211. doi:10.1038/s41586-025-08636-5 40011787 PMC13019327

[61] Zhang X, Zhang Y. 2009. Neural-immune communication in Caenorhabditis elegans. Cell Host Microbe 5:425–429. doi:10.1016/j.chom.2009.05.003 19454346

[62] Ryu KW, Fung TS, Baker DC, Saoi M, Park J, Febres-Aldana CA, Aly RG, Cui R, Sharma A, Fu Y, Jones OL, Cai X, Pasolli HA, Cross JR, Rudin CM, Thompson CB. 2024. Cellular ATP demand creates metabolically distinct subpopulations of mitochondria. Nature 635:746–754. doi:10.1038/s41586-024-08146-w 39506109 PMC11869630

