# Supplementary figures and images for: Microbial genetic screen identifies bacterial genes that compromise Caenorhabditis elegans reproductive fitness
Source: mSystems. 2026 May 7;11(6):e01698-25. doi: 10.1128/msystems.01698-25 (PMC13289084; doi:10.1128/msystems.01698-25)

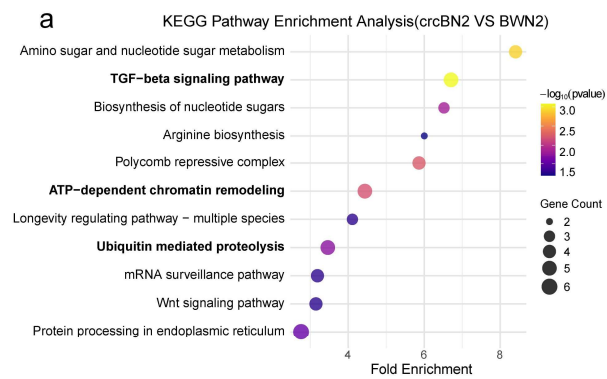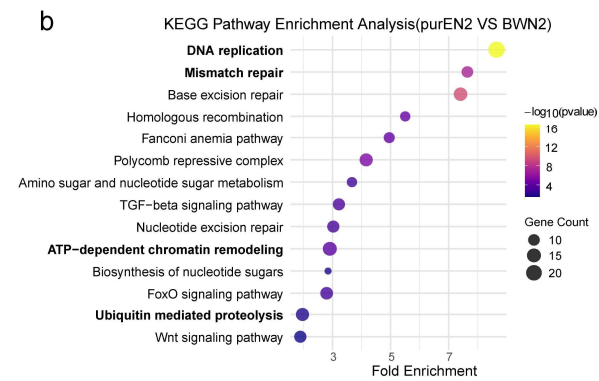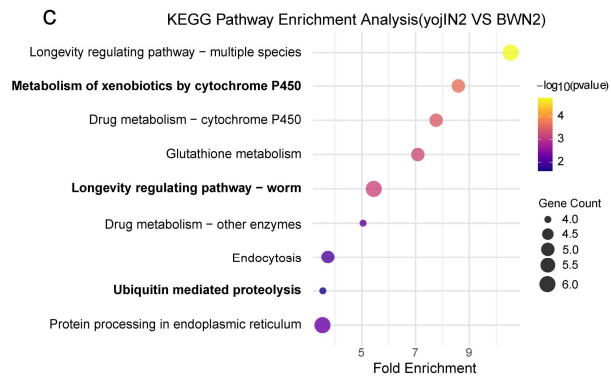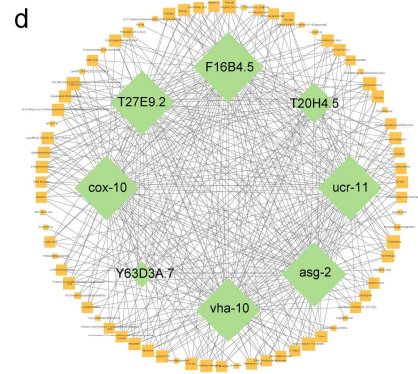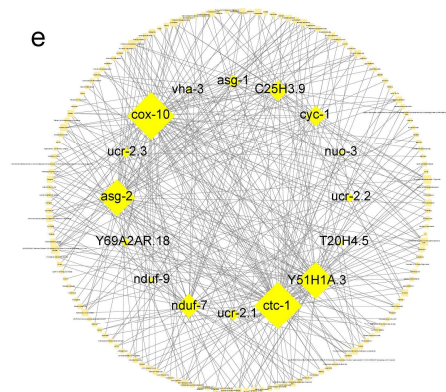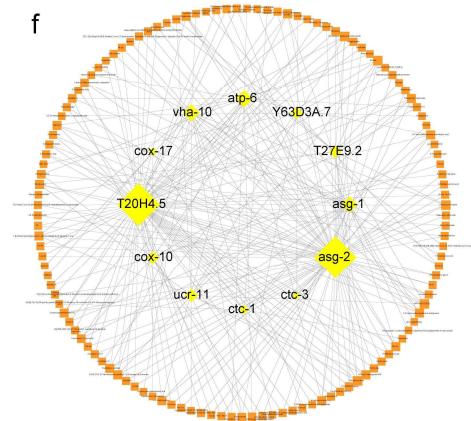

Supplement: Figure S2 — Differential gene KEGG pathway and gene-metabolite correlation analysis. [file msystems.01698-25-s0002.pdf]

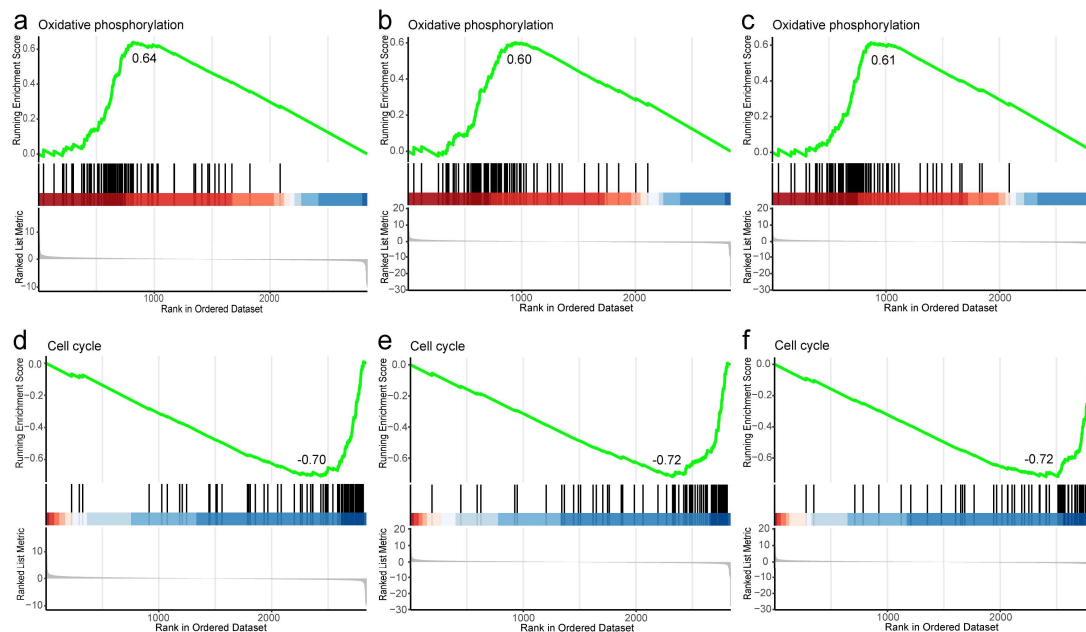

Supplement: Figure S3 — GSEA analysis of oxidative phosphorylation and cell cycle. [file msystems.01698-25-s0003.pdf]
