# Supplementary material for: Microbial genetic screen identifies bacterial genes that compromise Caenorhabditis elegans reproductive fitness
Source: mSystems. 2026 May 7;11(6):e01698-25. doi: 10.1128/msystems.01698-25 (PMC13289084; doi:10.1128/msystems.01698-25)
Supplement: Supplemental Legends — Legends for Figures S1-S3 and Tables S1-S6. [file msystems.01698-25-s0004.docx]

Figure S1

Analysis of three mutant strains of *E. coli* revealed their shared and unique differential proteins and metabolites, and constructed an interaction network of shared upregulated proteins. (a) Venn diagram of differentially expressed proteins among three *E. coli* mutant strains. (b) Venn diagram of differential metabolites among three *E. coli* mutant strains. (c) Protein–protein interaction network of the commonly up-regulated differential proteins in the three *E. coli* mutant strains.

Figure S2

Differential gene KEGG pathway and gene-metabolite correlation analysis of *C. elegans* fed with different mutant bacteria (*crcB*N2/*purE*N2/*yojI*N2) vs. control group (BWN2). (a-c) Bubble plot of KEGG pathways enriched by differential genes between (a)*crcB*N2 and BWN2, (b) *purE*N2 and BWN2, (c) *yojI*N2 and BWN2. (d-f) Gene-metabolite correlation network between (d)*crcB*N2 and BWN2, (e) *purE*N2 and BWN2, (f) *yojI*N2 and BWN2.

Figure S3

GSEA analysis of Oxidative phosphorylation and Cell cycle between *crcB*N2/*purE*N2/*yojI*N2 and BWN2 groups. (a-c) GSEA plots of oxidative phosphorylation: All genes were ranked by decreasing differential expression fold change, and genes are heavily enriched upstream. (a) *crcB*N2 VS BWN2: Normalized Enrichment Score (NES) = 2.342; p value = 5.33E-07; (b) *purE*N2 VS BWN2: Normalized Enrichment Score (NES) = 2.416; p value = 5.46E-08; (c) *yojI*N2 VS BWN2: Normalized Enrichment Score (NES) = 2.438; p value = 9.87E-08. (d-f) GSEA plots of cell cycle: All genes were ranked by decreasing differential expression fold change, and genes are heavily enriched downstream. (d) *crcB*N2 VS BWN2: Normalized Enrichment Score (NES) = -2.642; p value = 1.46E-09; (e) *purE*N2 VS BWN2: Normalized Enrichment Score (NES) = -2.677; p value = 2.65E-09; (f) *yojI*N2 VS BWN2: Normalized Enrichment Score (NES) = -2.536; p value = 3.76E-07.

Table S1

Specific information on the differential metabolites of the three mutant strains compared with wild-type *E.coli* (|log2FC| >0.5).

Table S2

In Caenorhabditis elegans fed with *ΔcrcB*, *ΔpurE*, and *ΔyojI* mutant bacteria, reproduction-related genes were statistically downregulated compared to the control group.

Table S3

Detailed information on the pathways and modules where the overall expression level of GSEA has undergone significant changes.

Table S4-S6

Transcriptomic and metabolomics data of *C. elegans* and proteomics and metabolomics data of *E. coli*.
