# Supplementary material for: Microbial genetic screen identifies bacterial genes that compromise Caenorhabditis elegans reproductive fitness
Source: mSystems. 2026 May 7;11(6):e01698-25. doi: 10.1128/msystems.01698-25 (PMC13289084; doi:10.1128/msystems.01698-25)
Supplement: Figure S1 — Differential proteins and metabolites and an interaction network of shared upregulated proteins. [file msystems.01698-25-s0001.pdf]

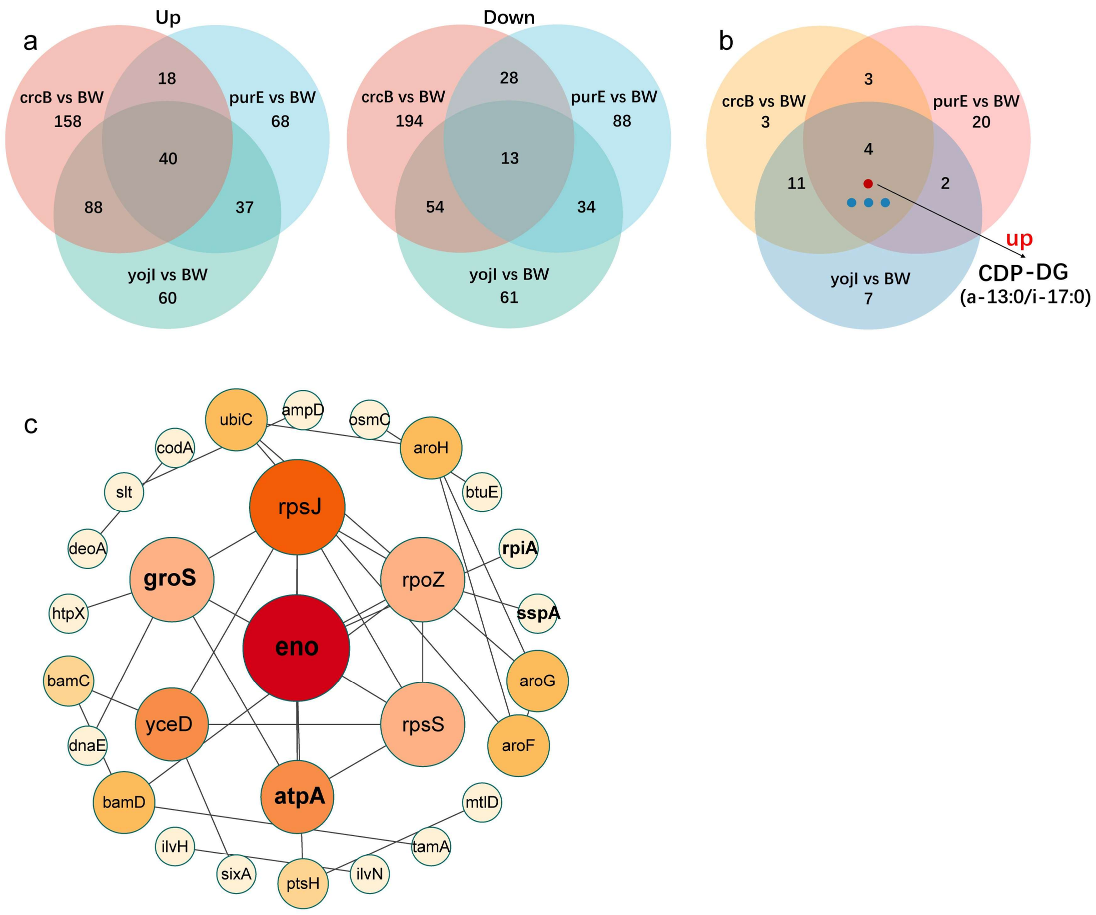
a
Up
crcB vs BW
158
18
purE vs BW
68
40
88
37
yojI vs BW
60
Down
crcB vs BW
194
28
purE vs BW
88
13
54
34
yojI vs BW
61
b
crcB vs BW
3
3
purE vs BW
20
4
11
2
yojI vs BW
7
up
CDP-DG
(a-13:0/i-17:0)
c
ubiC
ampD
osmC
aroH
codA
slt
btuE
rpsJ
deoA
rpiA
groS
rpoZ
htpX
sspA
eno
bamC
aroG
yceD
rpsS
dnaE
aroF
atpA
bamD
mtlD
ilvH
tamA
sixA
ptsH
ilvN
