# Supplementary material for: Microbial genetic screen identifies bacterial genes that compromise Caenorhabditis elegans reproductive fitness
Source: mSystems. 2026 May 7;11(6):e01698-25. doi: 10.1128/msystems.01698-25 (PMC13289084; doi:10.1128/msystems.01698-25)
Supplement: Reviewer comments [file reviewer-comments.pdf]

AMERICAN SOCIETY FOR MICROBIOLOGY | mSystems®

Microbial Genetic Screen Identifies Bacterial Genes that Compromise *Caenorhabditis elegans* Reproductive Fitness

Ziling Yang, Huigui Guo, Yudong Zhang, Xinzhou Jia, Yalun Wu, Tao Zhu, Ying Li, Jinyue Wang, Dianshuang Zhou, and Zuobin Zhu

Corresponding Author(s): Zuobin Zhu, Xuzhou Medical University

Review Timeline:

Submission Date:	December 1, 2025
Editorial Decision:	February 23, 2026
Revision Received:	March 18, 2026
Accepted:	March 31, 2026

Editor: Rupinder Kaur

Reviewer(s): Disclosure of reviewer identity is with reference to reviewer comments included in decision letter(s). The following individuals involved in review of your submission have agreed to reveal their identity: Tho Son Le (Reviewer #1)

Transaction Report:

(Note: With the exception of the correction of typographical or spelling errors that could be a source of ambiguity, letters and reports are not edited. The original formatting of letters and referee reports may not be reflected in this compilation.)

DOI: https://doi.org/10.1128/msystems.01698-25

Re: mSystems01698-25 (**Microbial Genetic Screen Identifies Bacterial Genes that Compromise *Caenorhabditis elegans* Reproductive Fitness**)

Dear Prof. Zuobin Zhu:

Thank you for the privilege of reviewing your work. Below you will find my comments, instructions from the mSystems editorial office, and the reviewer comments.

The reviewers agree that this work is potentially novel and offers valuable insights into microbiome-host interactions, particularly regarding microbial regulation of host reproductive biology. However, they also identified several important concerns regarding methodological transparency, interpretation of causality, and clarity of presentation that must be addressed to strengthen the rigor and impact of the manuscript. Therefore, I invite you to submit a major revision addressing all reviewer comments in detail. Please provide a comprehensive point-by-point response and clearly indicate where changes have been made in the manuscript.

Please return the manuscript within 60 days; if you cannot complete the modification within this time period, please contact me. If you do not wish to modify the manuscript and prefer to submit it to another journal, notify me immediately so that the manuscript may be formally withdrawn from consideration by mSystems.

Revision Guidelines
To submit your modified manuscript, log into the submission site at https://msystems.msubmit.net/cgi-bin/main.plex. Go to Author Tasks and click the appropriate manuscript title to begin. The information you entered when you first submitted the paper will be displayed; update this as necessary. Note the following requirements:

• Upload point-by-point responses to the issues raised by the reviewers in a file named "Response to Reviewers," NOT in your cover letter.
• Upload a compare copy of the manuscript (without figures) as a "Marked-Up Manuscript" file.
• Upload a clean .DOC/.DOCX version of the revised manuscript and remove the previous version.
• Each figure must be uploaded as a separate, editable, high-resolution file (TIFF or EPS preferred), and any multipanel figures must be assembled into one file.
• Any supplemental material intended for posting by ASM should be uploaded with their legends separate from the main manuscript. You can combine all supplemental material into one file (preferred) or split it into a maximum of 10 files with all associated legends included.

For complete guidelines on revision requirements, see our Submission and Review Process webpage. Submission of a paper that does not conform to guidelines may delay acceptance of your manuscript.

Data availability: ASM policy requires that data be available to the public upon online posting of the article, so please verify all links to sequence records, if present, and make sure that each number retrieves the full record of the data. If a new accession number is not linked or a link is broken, provide mSystems production staff with the correct URL for the record. If the accession numbers for new data are not publicly accessible before the expected online posting of the article, publication may be delayed; please contact production staff (mSystems@asmusa.org) immediately with the expected release date.

Publication Fees: For information on publication fees and which article types are subject to charges, visit our website. If your manuscript is accepted for publication and any fees apply, you will be contacted separately about payment during the production process; please follow the instructions in that e-mail. Arrangements for payment must be made before your article is published.

ASM Membership: Corresponding authors may join or renew ASM membership to obtain discounts on publication fees. Need to upgrade your membership level? Please contact Customer Service at Service@asmusa.org.

The ASM Journals program strives for constant improvement in our submission and publication process. Please tell us how we can improve your experience by taking this quick Author Survey.

Thank you for submitting your paper to mSystems.

Sincerely,
Rupinder Kaur
Editor
mSystems

Reviewer #3 (Comments for the Author):

This study presents a novel and systematic investigation into how microbial genetic variation regulates host reproductive fitness using an E. coli-C. elegans model. Grounded in microbial genetics with a comprehensive experimental design, this work offers a novel research approach for elucidating the molecular mechanisms underlying host reproductive disorders, holding both significant scientific value and promising exploratory potential. Nevertheless, several aspects would benefit from further clarification and revision to enhance the overall clarity, methodological rigor, and presentation quality.

1. Reproducibility of the "Worm Studio" Software
The manuscript states that progeny counting was performed using a self-developed software tool ("Worm Studio"), yet no reference, version number, algorithm description, or access link is provided.
For methodological transparency and reproducibility, the authors are encouraged to: Provide a citation if the software has been published; Include a GitHub or repository link if publicly available; Clarify version number and parameter settings.

2.Statistical Thresholds and Analytical Consistency
The RNA-seq section defines differentially expressed genes using "VIP > 1," which is typically derived from PLS-DA models and not from DESeq2 analysis. Since DESeq2 is based on a negative binomial generalized linear model, differential expression criteria should be derived from log2FoldChange and adjusted p-values (FDR).

3.Functional Context of ΔcrcB, ΔpurE, and ΔyojI
The manuscript identifies three bacterial mutants but does not sufficiently describe their known biological functions. Providing this context in the Introduction or early Results section would help readers understand why deletion of these functionally distinct genes may converge on similar metabolic phenotypes.

4.Unexplained Appearance of "27A6"
The Results section mentions "ΔcrcB, ΔpurE, ΔyojI, or 27A6 showed complete inhibition," yet no further information regarding 27A6 is provided.
This requires clarification. If 27A6 was not pursued further, the rationale for exclusion should be stated. Otherwise, this reference may cause confusion.

5.Strength of the "High Metabolism-Low Proliferation" Model
The central conclusion that bacterial perturbations induce a "high metabolism, low proliferation" host state is primarily supported by pathway enrichment analysis (GSEA and metabolite enrichment).
While suggestive, the current evidence remains correlative. The authors may consider: Explicitly acknowledging the model as inferential; Proposing future validation strategies (e.g., oxidative phosphorylation inhibition, cell-cycle modulation);
Tempering causal language in the Discussion would improve scientific balance.

6.Figure and Labeling Issues
Several figures contain typographical or formatting inconsistencies: Spelling errors in schematic diagrams (e.g., "Kieo collection"); Inconsistent reporting of p-values ("less than 0.0001" vs. star notation); Use of "ns" without statistical test specification.
A consistent statistical annotation scheme (* p<0.05, ** p<0.01, etc.) is recommended.

7.Supplementary Material Cross-Referencing
Some supplementary figures and tables are not clearly referenced in the main text. For example: Certain panels (e.g., Figure 6g-i) lack explicit citation; Table S1 should specify statistical thresholds (e.g., log2FC cutoff).
Ensuring systematic cross-referencing would improve readability.

8.Enrich the discussion section
The discussion section only mentioned the need for validation in complex microbial communities, but failed to analyze the possibility and limitations of the research results being transformed into higher organisms in light of the current research status of mammalian intestinal microbiota. It also did not clearly identify the specific potential targets for reproductive health intervention in the research findings. The depth and breadth of the discussion need to be enriched.

Reviewer #4 (Comments for the Author):

This manuscript presents an interesting and forward-looking investigation into the role of bacterial metabolites in C. elegans. The research provides in-depth insights into how microbial genetic variation influences complex host traits. Overall, this work offers a novel perspective for understanding host reproductive disorders from the standpoint of microbial genetics. Addressing the points below would significantly strengthen the manuscript.

1. The precise culture conditions of the E. coli variants used for feeding assays, such as the growth phase and optical density at harvest, have not been clearly specified.

2. Supplemental Method for Bacterial Heat-Inactivation Experiment.

3. What are the sampling methods for E. coli and Caenorhabditis elegans subjected to metabolomic and proteomic analyses?

4. Figure1 contains an unclear description: "Detailed flow chart of the schematic diagram in (a)." To improve precision, it is recommended to revise this to "Detailed screening workflow corresponding to the schematic in (a)."

5. Figure2 includes results from both inactivated and non-inactivated bacteria, but the difference between these conditions is not discernible from the figure.

6. Figure 3B, the number of samples analyzed per group is not clearly indicated.

7. The proposed causal link between bacterial CDP-DG accumulation and the upregulation of host oxidative phosphorylation is based primarily on correlative evidence. This limitation should be more explicitly acknowledged in the manuscript.

8. Expand the discussion on the translational implications of the study to clarify the potential reference value of the findings, and objectively point out the limitations of the simplified model of C. elegans fed with a single bacterial strain.

Dear Authors and Editors,

The manuscript presents an interesting study about the impact of the bacterial prey on the C. elegans N2 predator. MS used the high-throughput techniques to assess the metabolome, proteome, and transcriptome in both or either organisms under their interactions. In bacteria, the proteomic data was consistent with the metabolomic data such as of CDP-DG.

When C. elegans fed the bacterial mutants, it showed a tradeoff between low fecundity and extended lifespan in C. elegans. At the transcriptomic level, this shift revealed that the energy-producing pathways were increased, giving a longer lifespan and the cell cycle and nucleotide synthesis were disrupted, down-regulating less sperm production. However, from my point of view, the authors should provide more evidence.

Major review

1) Reference for “Untargeted metabolomics by LC-MS/MS” in Methods.
2) The catalog numbers of the machines and chemicals operated in the high-throughput techniques, maybe in a supplementary page.

Bacteria

3) Describing how the bacterial mutants were made even though they were outsourced.
4) Providing the information on the mutations within the bacteria, more important for the related genes. Although the authors mentioned in Discussion.
5) Providing the reference for “proteomic sequencing of E. coli” in Methods
6) Authors examined whether bacteria survived, died, and the stage of the cells (lysed or cellular) after pasteurization? The stage of tested bacteria may affect the nutrition of the tested C. elegans, leading to extended lifespan and low brood.
7) In Discussion, the authors cited heat-stable metabolites in which CDP-DG is heat-stable?

C. elegans

7) Describing how to process a conventional fluorescence microscope and ImageJ to assess the sperm nucleus number. Did the authors count nuclei in layers?

8) Did the worms acclimate to bacteria for generation(s) before the real tests on the respective bacteria? Explain why the authors did not acclimate worms to the bacteria, if the authors had reasons? Otherwise, I strongly suggest to do acclimation, at least for one experiment (fecundity, longevity, or metabolome). The reason is to “remove” the maternal effects that could interfere the transcriptomic and metabolomic data, and to “remove” bacteria that colonize in new plates from the transferred worms.

9) Describing how to set up the transcriptome sequencing?

10) To conclude the genetics of either energetic or spermatogenetic pathways in C. elegans regarding the metabolome of bacteria (like CDP-DG or others), the authors would use many mutants (accessible from CGC or particular laboratories) or test the mRNA with specific primers and/or protein expression in the wild-type C. elegans N2 <u>for at least one gene</u> to approve the

consistency of the transcriptomic data with metabolomic data. Instead, the authors may test on the GFP strain(s) in this scenario. Although the authors mentioned in Discussion.

11) What if the metabolites in C. elegans were contaminated by the bacteria that was in the intestine, the outer cuticle, and the vulval surrounding? Even using N2 and control BWN2.

Minor review

1000μL -> 1000 μL

minutes->min

Explain what "respective experimental conditions" are, at least one condition.

uniform size-> a uniform size

6 biological -> six biological

rewrite "To explore the mode of...of…of…"

"in the KEGG pathways[,]Citrate cycle (TCA cycle)[,]and Other…"

"Figure 6 Differential gene and metabolite[ ]enrichment analysis…"

abnormalities [34]? or abnormalities (Figure 6g-i)

Dear Editor and Reviewers,

We sincerely thank you for your time and effort in reviewing our manuscript. We greatly appreciate the constructive feedback, which has been invaluable in improving the quality of our work. In this revised version, we have carefully addressed all of the comments raised by the reviewers. We believe that the manuscript has been significantly strengthened and is now suitable for publication in mSystems. Below, we provide a detailed, point-by-point response to the reviewers' comments. All corresponding revisions have been incorporated into the manuscript and are highlighted accordingly.

Reviewer #3:

Comment 1. Reproducibility of the "Worm Studio" Software

The manuscript states that progeny counting was performed using a self-developed software tool ("Worm Studio"), yet no reference, version number, algorithm description, or access link is provided.

For methodological transparency and reproducibility, the authors are encouraged to: Provide a citation if the software has been published; Include a GitHub or repository link if publicly available; Clarify version number and parameter settings.

Response 1: We thank the reviewer for raising this important point regarding the reproducibility of our methodology. We have revised the "Determination of reproductive fitness" subsection of the Materials and Methods section to provide the necessary details. Specifically, since the core algorithm and validation of the "Worm Studio" software have been previously published in a dedicated methodology paper, we have now added a citation to this work in the revised manuscript.

We have added the citation in the revised manuscript:

After culturing at 20°C for 4 days, the number of progeny nematodes was automatically counted using Worm Studio[13]—an image analysis software independently developed by our research group—to improve statistical efficiency.

[13] Z. Jin, H. Yu, C. Haul, L. Wang, Z. Zhu, Q. Shen, X. Cao, WormTrack: Dataset and Benchmark for Multi-Object Tracking in Worm Crowds, Proceedings of the 31st ACM International Conference on Multimedia, 2023, pp. 5756-5763.

Comment 2. Statistical Thresholds and Analytical Consistency

The RNA-seq section defines differentially expressed genes using "VIP > 1," which is typically derived from PLS-DA models and not from DESeq2 analysis. Since DESeq2 is based on a negative binomial generalized linear model, differential expression criteria should be derived from log2FoldChange and adjusted p-values (FDR).

Response 2: We thank the reviewer for raising this critically important point and for providing such a precise correction. This was indeed a clerical error in our manuscript; we inadvertently applied metabolomics analysis criteria (VIP > 1) to the transcriptomics section, which is methodologically incorrect. In accordance with the reviewer's suggestion, we have now rigorously corrected this mistake. In the "RNA-seq analysis of *C. elegans*" subsection of the "Materials and Methods" section in the revised manuscript, the criteria for identifying differentially expressed genes have been revised to align with the standard conventions of DESeq2 analysis. This standard aligns with field conventions and ensures the reliability of the

analytical results.

The following modifications have been made in the manuscript:

Genes with a $|\log_2 FC| > 1$, and $P < 0.05$ were considered to have significantly different expression.” This standard aligns with field conventions and ensures the reliability of the analytical results.

Comment 3. Functional Context of *ΔcrcB*, *ΔpurE*, and *ΔyojI*

The manuscript identifies three bacterial mutants but does not sufficiently describe their known biological functions. Providing this context in the Introduction or early Results section would help readers understand why deletion of these functionally distinct genes may converge on similar metabolic phenotypes.

Response 3: Thank you for this valuable suggestion, which helps make the manuscript’s logic clearer. Providing the functional background of these three mutants upfront is important for readers to understand how three genes with different biological roles can produce similar phenotypes. We have added a brief description of their known functions immediately after the mutant screening in the Results section, along with corresponding references.

The following content has been added to the manuscript:

CrcB is implicated in cellular stress responses and membrane homeostasis under extreme conditions[19]. *PurE* encodes a key phosphoribosylaminoimidazole carboxylase essential for de novo purine biosynthesis[20, 21]. *YojI* encodes an ABC transporter likely involved in transmembrane transport[22].

[19] O. Sand, M. Gingras, N. Beck, C. Hall, N. Trun, Phenotypic characterization of overexpression or deletion of the Escherichia coli crcA, cspE and crcB genes, Microbiology (Reading) 149(Pt 8) (2003) 2107-2117.

[20] E. Meyer, N.J. Leonard, B. Bhat, J. Stubbe, J.M. Smith, Purification and characterization of the purE, purK, and purC gene products: identification of a previously unrecognized energy requirement in the purine biosynthetic pathway, Biochemistry 31(21) (1992) 5022-32.

[21] W. Watanabe, G. Sampei, A. Aiba, K. Mizobuchi, Identification and sequence analysis of Escherichia coli purE and purK genes encoding 5'-phosphoribosyl-5-amino-4-imidazole carboxylase for de novo purine biosynthesis, J Bacteriol 171(1) (1989) 198-204.

[22] M.A. Delgado, P.A. Vincent, R.N. Farias, R.A. Salomon, YojI of Escherichia coli functions as a microcin J25 efflux pump, J Bacteriol 187(10) (2005) 3465-70.

Comment 4. Unexplained Appearance of “27A6”

The Results section mentions “*ΔcrcB*, *ΔpurE*, *ΔyojI*, or 27A6 showed complete inhibition,” yet no further information regarding 27A6 is provided.

This requires clarification. If 27A6 was not pursued further, the rationale for exclusion should be stated. Otherwise, this reference may cause confusion.

Response 4: This is a very important point raised by the reviewer, and we thank you for highlighting this ambiguity in our writing. The reason for excluding 27A6 is as follows: Although 27A6 exhibited significant phenotypic inhibition during the preliminary screening, there are currently no studies on the annotation or functional characteristics of this gene. In this study, our mechanistic analyses are based on known gene functions; including 27A6 in subsequent experiments would render certain parts of the results unexplained. Therefore, we

prefer to select mutants with known characteristics to obtain interpretable results. To avoid any misunderstanding for readers, we have clearly stated the exclusion criteria in the "High-throughput screen identifies *E. coli* single-gene deletion mutants that inhibit *C. elegans* reproduction" subsection of the "Results".

The manuscript has been modified as follows:

CrcB is implicated in cellular stress responses and membrane homeostasis under extreme conditions[19]. *PurE* encodes a key phosphoribosylaminoimidazole carboxylase essential for de novo purine biosynthesis[20, 21]. *YojI* encodes an ABC transporter likely involved in transmembrane transport[22]. The mutant strain 27A6 also completely inhibited progeny production in the primary screen but lacks an annotated gene and functional characterization. To ensure reliability in subsequent mechanistic studies, only *ΔcrcB*, *ΔpurE*, and *ΔyojI* were selected for further analysis.

Comment 5. Strength of the "High Metabolism–Low Proliferation" Model

The central conclusion that bacterial perturbations induce a "high metabolism, low proliferation" host state is primarily supported by pathway enrichment analysis (GSEA and metabolite enrichment).

While suggestive, the current evidence remains correlative. The authors may consider: Explicitly acknowledging the model as inferential; Proposing future validation strategies (e.g., oxidative phosphorylation inhibition, cell-cycle modulation);

Tempering causal language in the Discussion would improve scientific balance.

Response 5: Thank you for your feedback which helped us improve our interpretation of the results. We have revised the overly absolute statements in the "Discussion" section to "The omics data strongly suggest that these three mutants may induce a 'high energy consumption and low proliferation' state in the host by disrupting host oxidative phosphorylation as well as cell cycle and DNA replication", in order to more accurately reflect the correlation of our research results. We also clearly specified the direction for future experimental verification, such as through inhibitors or RNAi to inhibit oxidative phosphorylation Additionally, to support the new discussion points, we have cited two references.

We have made the following revisions to the manuscript:

Although the original gene functions of the three mutant strains differ (involved in stress response, purine synthesis, and unknown function, respectively)[19, 43], the omics data strongly suggest that these three mutants may induce a "high energy consumption and low proliferation" state in the host by disrupting host oxidative phosphorylation as well as cell cycle and DNA replication. We hypothesize that metabolic stress caused by bacterial gene deletion (possibly due to insufficient nutrient supply or accumulation of harmful metabolites) forces the host to enhance energy production to maintain basic homeostasis. However, this compensatory response is costly, leading to the redistribution of energy and raw materials originally used for the rapid division of germ cells, thereby resulting in decreased reproductive fitness. This mechanism is highly consistent with the "reproduction-lifespan trade-off" theory: when organisms perceive environmental stress, they prioritize somatic maintenance and stress defense, sacrificing reproductive investment[44-46]. This also reasonably explains the phenomenon of extended *C. elegans* lifespan observed in this study. However, the current evidence remains correlative and requires further research for validation.

For example, *C. elegans* could be treated with specific inhibitors of oxidative phosphorylation, such as rotenone[47], to test whether they can suppress the mutant-induced reproductive phenotype. Alternatively, RNAi-mediated knockdown of key cell cycle genes could be employed to investigate their direct relationship with the observed reduction in fecundity[48].

[47] N. Li, K. Ragheb, G. Lawler, J. Sturgis, B. Rajwa, J.A. Melendez, J.P. Robinson, Mitochondrial complex I inhibitor rotenone induces apoptosis through enhancing mitochondrial reactive oxygen species production, J Biol Chem 278(10) (2003) 8516-25.

[48] N.D. Sanscrainte, H. Arimoto, C.M. Waits, L.Y. Li, D. Johnson, C. Geden, J.J. Becnel, A.S. Estep, Reduction in Musca domestica fecundity by dsRNA-mediated gene knockdown, PLoS One 13(1) (2018) e0187353.

Comment 6. Figure and Labeling Issues

Several figures contain typographical or formatting inconsistencies: Spelling errors in schematic diagrams (e.g., "Kieo collection"); Inconsistent reporting of p-values ("less than 0.0001" vs. star notation); Use of "ns" without statistical test specification.

A consistent statistical annotation scheme (*$P < 0.05$, **$P < 0.01$, etc.) is recommended.

Response 6: We thank the reviewer for their meticulous review. We have thoroughly checked and corrected all figures and annotations throughout the manuscript. We have corrected "Kieo collection" to "Keio collection" in Figure 1b. We have standardized the statistical annotations across all figure legends. All bar charts now consistently follow the notation: *$P < 0.05$, **$P < 0.01$, ***$P < 0.001$, ****$P < 0.0001$. Where we previously wrote "$P < 0.0001$," we have retained the exact p-value alongside the "****" notation, balancing rigor with visual clarity. In the legend for Figure 3C, we have explicitly explained the meaning of "ns," i.e., "ns" indicates no statistically significant effect.

We have updated the text and figures in the revised manuscript:

1. Dunnett's test was applied to compare all treatment groups with the control group. *$P < 0.05$, **$P < 0.01$, ***$P < 0.001$, ****$P < 0.0001$, "ns" indicates no statistically significant effect. The survival curves of *C. elegans* were analyzed using the Log-rank (Mantel-Cox) test.

2. (c) Number of progeny produced by *C. elegans* when fed with 8 selected *E. coli* mutants of interest. Statistical significance was determined using Dunnett's test compared to the BW25113 control group. *$P < 0.05$, **$P < 0.01$, ***$P < 0.001$, and ****$P < 0.0001$.

3. (a) Gene mutations in *E. coli* may significantly inhibit the progeny number of *C. elegans*. Comparison of progeny numbers between the control group and three mutant groups (*ΔcrcB*, *ΔpurE*, *ΔyojI*). ****$P < 0.0001$.

4. (d) Progeny number of *C. elegans* on the first day when fed with inactivated *E. coli* (*ΔcrcB*^{i}, *ΔpurE*^{i}, *ΔyojI*^{i}) and BW25113(control^i). Statistical significance was determined using Dunnett's test compared to the BW25113 control group. *$P < 0.05$, **$P < 0.01$.

5. Figure 3 Effects of *E. coli* mutants on sperm characteristics and survival of *C. elegans*. (a) DAPI staining of *C. elegans* fed with BW25113 and three mutant strains. The blue-fluorescent particles indicated by white arrows are sperm. (b) Counting of *C. elegans* sperm under a fluorescence microscope using ImageJ. ****$P < 0.0001$.

6. Figure 1

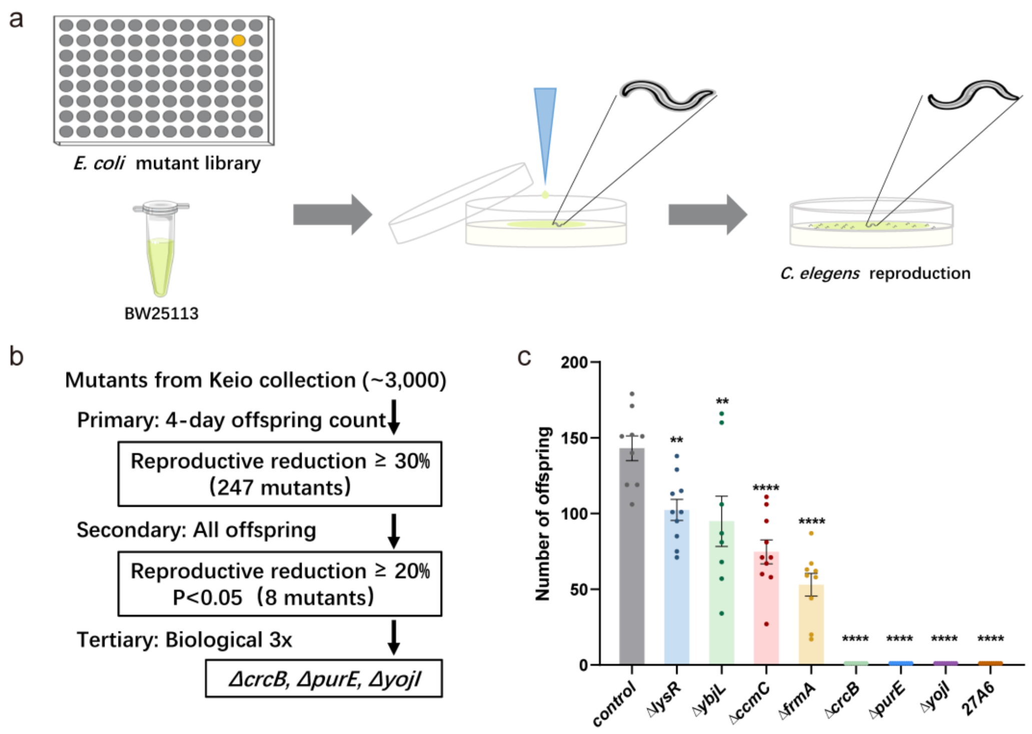

Comment 7. Supplementary Material Cross-Referencing

Some supplementary figures and tables are not clearly referenced in the main text. For example: Certain panels (e.g., Figure 6g–i) lack explicit citation; Table S1 should specify statistical thresholds (e.g., log2FC cutoff).

Ensuring systematic cross-referencing would improve readability.

Response 7: Thank you for pointing this out. We have carefully reviewed the entire manuscript to ensure that every supplementary figure and table is accurately mentioned in the appropriate place within the main text. In the "Results" section describing the "*E. coli* Mutants Induce *C. elegens* to Enter a 'High Metabolism, Low Proliferation' Metabolic State" findings, statement "Among them, core energy-producing pathways such as the Citrate cycle (TCA cycle), Glutathione metabolism, and Pyruvate metabolism were significantly enriched" corresponds to Figure 6g-i. For statistical thresholds for Table S1, to improve transparency, we have added a new column displaying $|log_2FC|$ values in Supplementary Table S1.

We have made the following modifications:

1. Among them, core energy-producing pathways such as the Citrate cycle (TCA cycle), Glutathione metabolism, and Pyruvate metabolism were significantly enriched (Figure 6g-i), indicating an upregulation of the host's overall energy metabolism level.

2. Supplementary Table S1

	Metabolite	Vip	pvalue	fdr	$\|log_2FC\|$	up/down
	Glaucine	5.570235825	0.0005187	0.01408	2.496818615	up
	Iclaprim	3.86263104	0.03523	0.1287	1.626543716	up
	CDP-DG(a-13:0/i-17:0)	4.044248921	0.02448	0.1011	0.695685282	up
	Proscillaridin	3.592514791	0.02913	0.1132	0.589729493	down
	LysoPG(18:2(9Z,12Z)/0:0)	4.723968913	0.0001294	0.006388	0.590599135	down
	Pe(22:1/0:0)	5.200810329	0.0000786	0.00455	0.597197953	down
	Fexofenadine	4.763206225	0.0003369	0.01096	0.600780929	down
	Eicosapentaenoic acid	4.85311823	0.0001911	0.007656	0.60650991	down
	Lpc(18:1)	5.728218448	0.0001671	0.007231	0.615534527	down
	LysoPE(20:5(5Z,8Z,11Z,14Z,17Z)/0:0)	4.918143551	0.004097	0.04928	0.658272265	down
crcB_vs_BW	Met-Abu-OH	4.379044202	0.0007872	0.01791	0.761727624	down
	Lpe(18:2)	4.537788305	0.015	0.08011	0.848433965	down
	'anandamide (20:5, n-3)'	5.47886459	0.0002341	0.008761	0.856283644	down

Table S1

Specific information on the differential metabolites of the three mutant strains compared with wild-type *E.coli* (|log2FC|>0.5).

Comment 8. Enrich the discussion section

The discussion section only mentioned the need for validation in complex microbial communities, but failed to analyze the possibility and limitations of the research results being transformed into higher organisms in light of the current research status of mammalian intestinal microbiota. It also did not clearly identify the specific potential targets for reproductive health intervention in the research findings. The depth and breadth of the discussion need to be enriched.

Response 8: We thank the reviewer for this suggestion, which helped us expand our discussion on the broader biological significance and translational potential of our findings. Accordingly, we have substantially expanded the “Discussion” section by revising the final paragraph to include the following two aspects: a systematic analysis of the potential and challenges of translating our findings to higher organisms (such as mammals); and the identification of several specific potential targets for reproductive health intervention based on our findings. In addition, to support the newly added discussion points, we have incorporated several new references.

The following paragraph has been added to the manuscript:

1. When considering the translation of these findings to higher organisms such as mammals, this study offers a valuable theoretical framework while also facing significant challenges. On one hand, the core metabolic pathways implicated here, including oxidative phosphorylation, purine metabolism, and glycerophospholipid metabolism, are evolutionarily conserved[57-59]. This suggests that dysregulation of homologous genes in the human gut microbiota could potentially affect host reproductive health through similar mechanisms, providing a proof-of-principle for understanding the molecular basis of reproductive disorders such as polycystic ovary syndrome. On the other hand, mammals possess far more complex neuro-endocrine-immune networks than *C. elegans*, through which the gut microbiota may exert indirect effects on reproduction[60, 61]. The direct metabolite-host interaction model observed in this study may therefore be modulated or overridden by other regulatory signals in higher organisms.

[57] G. Barshad, A. Blumberg, T. Cohen, D. Mishmar, Corrigendum: Human primitive brain

displays negative mitochondrial-nuclear expression correlation of respiratory genes, Genome Res 32(8) (2022) 1626.
[58] E. Sendinc, Y. Shi, RNA m6A methylation across the transcriptome, Mol Cell 83(3) (2023) 428-441.
[59] N. Senoo, D.K. Chinthapalli, M.G. Baile, V.K. Golla, B. Saha, A.O. Oluwole, O.B. Ogunbona, J.A. Saba, T. Munteanu, Y. Valdez, K. Whited, M.S. Sheridan, D. Chorev, N.N. Alder, E.R. May, C.V. Robinson, S.M. Claypool, Functional diversity among cardiolipin binding sites on the mitochondrial ADP/ATP carrier, EMBO J 43(14) (2024) 2979-3008.
[60] X. Li, H. Zhang, T. Hodder, W. Wang, C.L. Myers, L.S. Yilmaz, A.J.M. Walhout, Systems-level design principles of metabolic rewiring in an animal, Nature 640(8057) (2025) 203-211.
[61] X. Zhang, Y. Zhang, Neural-immune communication in Caenorhabditis elegans, Cell Host Microbe 5(5) (2009) 425-9.
2. Despite these limitations, this study identifies potential targets for reproductive health intervention: CDP-DG accumulation may serve as a biomarker for microbial-mediated host metabolic stress; the imbalance between oxidative phosphorylation and cell cycle gene expression suggests the value of modulating host energy metabolism[62]; and most importantly, this work opens a new avenue for intervention along the microbe–host metabolic axis. Future strategies could indirectly protect host reproductive function by reshaping microbial metabolism–for example, by reducing CDP-DG production.
[62] K.W. Ryu, T.S. Fung, D.C. Baker, M. Saoi, J. Park, C.A. Febres-Aldana, R.G. Aly, R. Cui, A. Sharma, Y. Fu, O.L. Jones, X. Cai, H.A. Pasolli, J.R. Cross, C.M. Rudin, C.B. Thompson, Cellular ATP demand creates metabolically distinct subpopulations of mitochondria, Nature 635(8039) (2024) 746-754.

Reviewer #4:

Comment 1. The precise culture conditions of the *E. coli* variants used for feeding assays, such as the growth phase and optical density at harvest, have not been clearly specified.

Response 1: We fully agree that precise culture conditions are critical for experimental reproducibility. Following your suggestion, we have added the specific culture conditions for the *E. coli* strains used in the *C. elegans* feeding assays to the “Bacterial strains: *E. coli* single-gene knockout strains” subsection of the “Materials and Methods”.

We have made the following modification in the manuscript:

Prior to use, *E. coli* strains were cultured with shaking in Luria-Bertani (LB) medium at 37°C and 200 rpm until reaching the logarithmic growth phase (OD600 of 0.6-0.8). The bacterial culture was then pelleted by centrifugation at 7000 rpm for 5 minutes; after discarding the supernatant, the bacterial pellet was resuspended in 1000μL of fresh LB medium, and 100μL of the resuspended culture was transferred onto NGM plates for subsequent *C. elegans*

feeding and experimental assays.

Comment 2. Supplemental Method for Bacterial Heat-Inactivation Experiment.

Response 2: We thank the reviewer for this valuable suggestion. We agree that providing a detailed protocol for the heat-inactivation experiment is essential for reproducibility. In response, we have added a dedicated subsection in the Materials and Methods section to describe this procedure in full.

The following paragraph has been incorporated into the manuscript:

Bacterial heat-inactivation assay

To determine whether the reproductive inhibitory effect of the mutant strains required live bacterial metabolism, *E. coli* strains (BW25113, *ΔcrcB*, *ΔpurE*, and *ΔyojI*) were subjected to heat inactivation. Bacterial cultures were grown to logarithmic phase as described above. The cultures were then placed in a 65°C water bath for 30 minutes. After heat treatment, 100 μL of the inactivated bacterial suspension was spread onto LB agar plates and incubated overnight at 37°C to verify complete inactivation (no colony formation). Only batches showing no growth were used for subsequent *C. elegans* feeding assays. The inactivated bacteria were then spread onto NGM plates, and *C. elegans* reproductive fitness was assessed as described in the "Determination of reproductive fitness" section.

Comment 3. What are the sampling methods for *E. coli* and Caenorhabditis elegans subjected to metabolomic and proteomic analyses?

Response 3: We thank the reviewer for this important question. We recognize that the original manuscript lacked detailed descriptions of the sampling methods for both *E. coli* and *C. elegans* used in the omics analyses. This omission could hinder the reproducibility of our experiments and obscure the interpretation of the multi-omics data, as sampling conditions (e.g., growth phase, harvest timing, washing steps) can significantly influence metabolite and protein profiles.

To address this issue, in the "Proteomic sequencing of *E. coli*" section, we have supplemented the collection method of *E. coli* before sequencing, and in the "Untargeted metabolomics by LC-MS/MS" section, we have supplemented the comprehensive collection method of *C. elegans*.

We have revised the manuscript as follows:

1. Proteomic sequencing of *E. coli*

For proteomic and metabolomic analyses, *E. coli* strains (BW25113, *ΔcrcB*, *ΔpurE*, *ΔyojI*) were cultured in LB medium to logarithmic phase (OD600 of 0.6-0.8). Bacterial cells were harvested by centrifugation at 7000 rpm for 5 minutes at 4°C. The supernatant was discarded, and the bacterial pellet was washed three times with ice-cold phosphate-buffered saline (PBS). The pellet was then flash-frozen in liquid nitrogen and stored at -80°C until further processing. Six biological replicates were collected per group. The samples were taken out in a frozen state, and protein lysis buffer was added.

2. Untargeted metabolomics by LC-MS/MS

For metabolomic analysis of *C. elegans*, synchronized L1-stage larvae were fed the respective *E. coli* strains (BW25113, *ΔcrcB*, *ΔpurE*, *ΔyojI*) on NGM plates at 20°C. Young adult worms (approximately 24 hours post-L4 stage) were collected. Worms were washed three times with

M9 buffer to remove residual bacteria, allowed to settle by gravity, and the supernatant was aspirated. The clean worm pellets were flash-frozen in liquid nitrogen and stored at -80°C. Each group collected six biological replicate samples, and the total volume of each sample was approximately 0.5 ml. The experiment was performed using a Thermo Fisher UHPLC-Q Exactive HF-X system coupled with an HSS T3 column (100 mm × 2.1 mm, 1.8 µm).

Comment 4. Figure1 contains an unclear description: "Detailed flow chart of the schematic diagram in (a)." To improve precision, it is recommended to revise this to "Detailed screening workflow corresponding to the schematic in (a)."

Response 4: We sincerely thank the reviewer for this meticulous observation and for providing a specific suggestion to enhance the clarity and precision of our figure legend. We agree that the original phrasing, "Detailed flow chart of the schematic diagram in (a)," was ambiguous and could potentially confuse readers by not clearly distinguishing between the conceptual setup in (a) and the procedural steps in (b). Therefore, we have modified the description of Figure 1b to "(b) The detailed screening workflow corresponding to the schematic in (a)".

We have made the corrections in the manuscript:

Figure 1 Identification of *E. coli* mutants that delay *C. elegans* fertility. (a) Schematic diagram of the high-throughput screening concept, in which *C. elegans* were fed with *E. coli* mutants from the Keio collection. (b) Detailed screening workflow corresponding to the schematic in (a). Three biological replicates were performed, and three mutants (*ΔcrcB*, *ΔpurE*, *ΔyojI*) were finally identified. The entire process reflects the stepwise screening to determine mutants associated with a specific phenotype (reduced reproductive fitness).

Comment 5. Figure2 includes results from both inactivated and non-inactivated bacteria, but the difference between these conditions is not discernible from the figure.

Response 5: We thank the reviewer for pointing this out. We agree that the original figure lacked clarity in distinguishing between live and inactivated bacterial experiments, which may have caused confusion. Without altering the overall structure of the figure, we have revised Figure 2 as follows to improve its clarity: First, in Figure 2d, we adjusted the colors of the bars representing the heat-inactivated bacterial experiments, using darker colors to create a clear visual contrast. Additionally, we introduced the notation $\Delta crcB^{i}$, $\Delta purE^{i}$, $\Delta yojI^{i}$, and control^{i} (where "I" denotes inactivated), allowing readers to distinguish live and inactivated bacteria at a glance. Correspondingly, we have revised the Figure 2 legend. The legend is now clearly divided into two sections: (a-b) Live bacterial experiments and (c-d) Heat-inactivated bacterial experiments, with an explanation that inactivated groups are denoted with a superscript "I". We believe these revisions have effectively enhanced the distinction and understanding of the results from live versus inactivated bacterial experiments.

We have updated the text and figures in the revised manuscript:

1. Figure 2 *E. coli* mutants inhibited *C. elegans* reproduction both before and after inactivation. (a-b) Live bacterial experiments. When fed with live bacteria, *E. coli* gene mutations significantly inhibited the progeny number of *C. elegans*. (a) Gene mutations in *E. coli* may significantly inhibit the progeny number of *C. elegans*. Comparison of progeny numbers between the control group and three mutant groups (*ΔcrcB*, *ΔpurE*, *ΔyojI*) . ****P <

0.0001. (b) Changes in progeny number (reproductive cycle) of *C. elegans* fed with BW25113 and *E. coli* mutant strains (*ΔcrcB*, *ΔpurE*, *ΔyojI*) over a 12-day period. (c-d) Heat-inactivated bacterial experiments. To investigate whether this effect depends on live bacterial metabolism, bacteria were heat-inactivated at 65°C for 30 minutes prior to feeding assays (inactivated groups are denoted with a superscript "i"). (c) Representative images of progeny produced by individual *C. elegans* on the first day when fed with inactivated *E. coli* ($\Delta crcB^{i}$, $\Delta purE^{i}$, $\Delta yojI^{i}$) and BW25113(control^{i}). (d) Progeny number of *C. elegans* on the first day when fed with inactivated *E. coli* ($\Delta crcB^{i}$, $\Delta purE^{i}$, $\Delta yojI^{i}$) and BW25113(control^{i}). Statistical significance was determined using Dunnett's test compared to the BW25113 control group. **P* < 0.05, ***P* < 0.01.

2. Figure 2

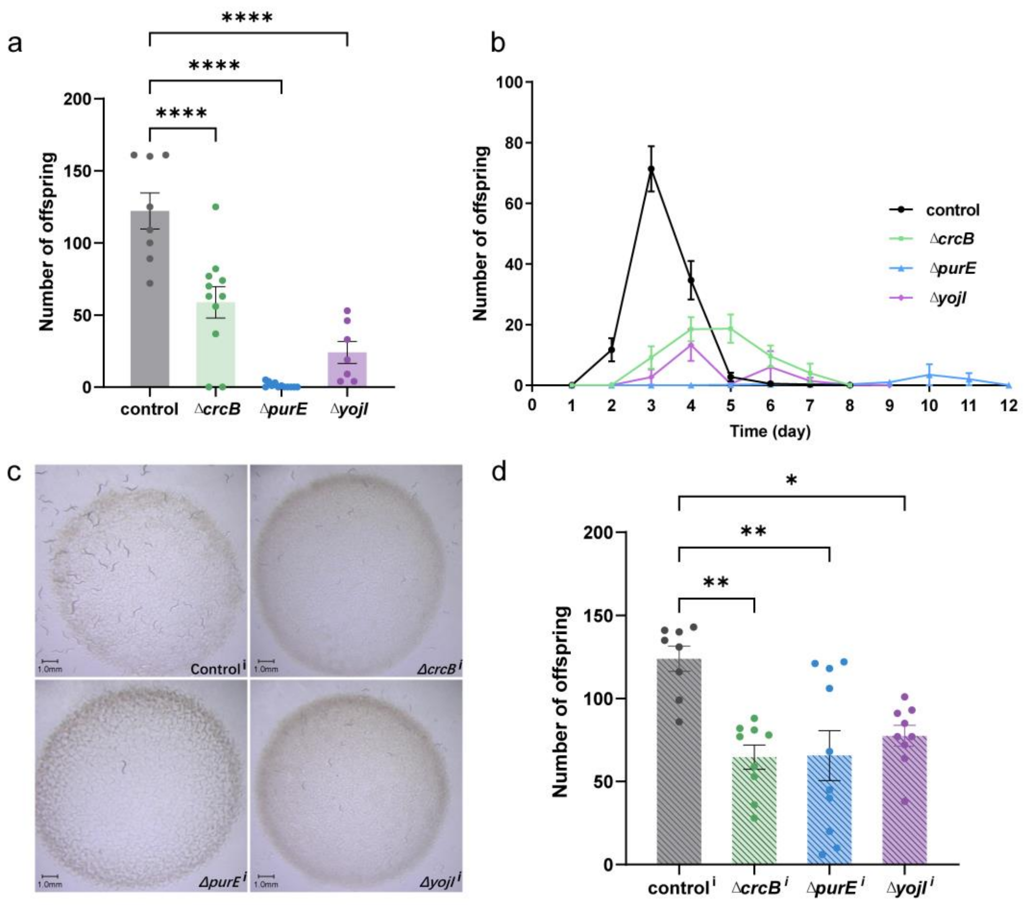

Comment 6. Figure 3B, the number of samples analyzed per group is not clearly indicated.

Response 6: We thank the reviewer for pointing out this omission. We agree that clearly indicating the sample size is essential for data transparency and reproducibility. To address this, we have now explicitly added the sample size to the legend of Figure 3b.

We have made the following revisions to the manuscript:

Figure 3 Effects of *E. coli* mutants on sperm characteristics and survival of *C. elegans*. (a) DAPI staining of *C. elegans* fed with BW25113 and three mutant strains. The

blue-fluorescent particles indicated by white arrows are sperm. (b) Counting of *C. elegans* sperm under a fluorescence microscope using ImageJ. ****P < 0.0001. (c) Comparison of motility data (vertical axis represents the number of head swings) between the *ΔcrcB*, *ΔpurE*, *ΔyojI* groups and the control group. The unit is the movement value per 30 seconds. “ns” indicates no statistically significant effect. (d) Survival curves of *C. elegans* fed with *ΔcrcB*, *ΔpurE*, *ΔyojI* and the control group fed with BW25113 (BW25113, n=204; *ΔcrcB*, n=171; *ΔpurE*, n=136; *ΔyojI*, n =164).

Comment 7. The proposed causal link between bacterial CDP-DG accumulation and the upregulation of host oxidative phosphorylation is based primarily on correlative evidence. This limitation should be more explicitly acknowledged in the manuscript.

Response 7: We thank the reviewer for raising this critical point, which accurately identifies an important nuance in the interpretation of our findings. We fully agree that the relationship between bacterial CDP-DG accumulation and host oxidative phosphorylation upregulation, as currently presented, is inferred from correlative multi-omics integration rather than established through direct causal interrogation. In response to this comment, we have now more explicitly addressed this limitation in the “Discussion” section. Specifically, we have added a paragraph that acknowledges the correlative nature of this link, discusses the inherent challenges in establishing causality in host–microbiota metabolic crosstalk studies, and outlines concrete experimental strategies that would be required to validate the proposed mechanism.

The following sentences have been added to the manuscript:

This study is based on a simplified model system in which *C. elegans* was fed a single bacterial strain (*E. coli*). While this approach facilitates the precise dissection of causal relationships between individual microbial genes and host phenotypes under controlled conditions, it cannot fully recapitulate the complexity of a multi-kingdom gut microbial community in nature. In a real ecosystem, interactions such as metabolic cross-feeding and competitive exclusion among microbes may potentiate or mask the effects observed with a single mutant strain[55, 56]. Furthermore, the proposed link between bacterial CDP-DG accumulation and the upregulation of host oxidative phosphorylation is currently supported primarily by correlative evidence from multi-omics integration. Direct functional validation, such as exogenous CDP-DG supplementation to *C. elegans* fed control bacteria or genetic manipulation of bacterial CDP-DG synthesis pathways, is required to establish causality.

Comment 8. Expand the discussion on the translational implications of the study to clarify the potential reference value of the findings, and objectively point out the limitations of the simplified model of *C. elegans* fed with a single bacterial strain.

Response 8: We sincerely thank the reviewer for this highly insightful suggestion. This comment helped us recognize that our initial manuscript lacked sufficient discussion regarding both the translational implications of our findings and the limitations of our model system. Accordingly, we have added two new paragraphs to the “Discussion”. The first addresses the inherent limitations of using a single bacterial strain in *C. elegans*, noting that natural gut ecosystems involve multi-kingdom microbial interactions that may modulate the observed effects. The second discusses the translational relevance of our findings, proposing

CDP-DG as a potential biomarker and highlighting the therapeutic promise of targeting microbial metabolism–such as engineering bacteria to reduce CDP-DG production–as an indirect strategy to preserve host reproductive health. To maintain logical coherence, we have inserted the discussions on “clarifying the potential reference value of the findings” and “the limitations of the simplified model of *C. elegans* fed with a single bacterial strain” into separate paragraphs.

The following text has been added to the manuscript:

1. This study is based on a simplified model system in which *C. elegans* was fed a single bacterial strain (*E. coli*). While this approach facilitates the precise dissection of causal relationships between individual microbial genes and host phenotypes under controlled conditions, it cannot fully recapitulate the complexity of a multi-kingdom gut microbial community in nature. In a real ecosystem, interactions such as metabolic cross-feeding and competitive exclusion among microbes may potentiate or mask the effects observed with a single mutant strain[55, 56]. Furthermore, the proposed link between bacterial CDP-DG accumulation and the upregulation of host oxidative phosphorylation is currently supported primarily by correlative evidence from multi-omics integration. Direct functional validation, such as exogenous CDP-DG supplementation to *C. elegans* fed control bacteria or genetic manipulation of bacterial CDP-DG synthesis pathways, is required to establish causality.

[55] E.J. Culp, A.L. Goodman, Cross-feeding in the gut microbiome: Ecology and mechanisms, Cell Host Microbe 31(4) (2023) 485-499.

[56] K.Z. Coyte, J. Schluter, K.R. Foster, The ecology of the microbiome: Networks, competition, and stability, Science 350(6261) (2015) 663-6.

2. Despite these limitations, this study identifies potential targets for reproductive health intervention: CDP-DG accumulation may serve as a biomarker for microbial-mediated host metabolic stress; the imbalance between oxidative phosphorylation and cell cycle gene expression suggests the value of modulating host energy metabolism[62]; and most importantly, this work opens a new avenue for intervention along the microbe–host metabolic axis. Future strategies could indirectly protect host reproductive function by reshaping microbial metabolism–for example, by reducing CDP-DG production.

[62] K.W. Ryu, T.S. Fung, D.C. Baker, M. Saoi, J. Park, C.A. Febres-Aldana, R.G. Aly, R. Cui, A. Sharma, Y. Fu, O.L. Jones, X. Cai, H.A. Pasolli, J.R. Cross, C.M. Rudin, C.B. Thompson, Cellular ATP demand creates metabolically distinct subpopulations of mitochondria, Nature 635(8039) (2024) 746-754.

Once again, we thank you for your time and diligent effort. We believe that the manuscript, revised according to your valuable comments, has been significantly improved in terms of scientific rigor, logical clarity, and readability. We sincerely hope that the revised manuscript is now suitable for publication in mSystems.

Yours sincerely,

Corresponding Authors: Zuobin Zhu

Affiliation: Xuzhou Medical University

Date: May 11, 2026

Re: mSystems01698-25R1 (**Microbial Genetic Screen Identifies Bacterial Genes that Compromise *Caenorhabditis elegans* Reproductive Fitness**)

Dear Prof. Zuobin Zhu:

Your manuscript has been accepted, and I am forwarding it to the ASM production staff for publication. Your paper will first be checked to make sure all elements meet the technical requirements. ASM staff will contact you if anything needs to be revised before copyediting and production can begin. Otherwise, you will be notified when your proofs are ready to be viewed.

Data Availability: ASM policy requires that data be available to the public upon online posting of the article, so please verify all links to sequence records, if present, and make sure that each number retrieves the full record of the data. If a new accession number is not linked or a link is broken, provide production staff with the correct URL for the record. If the accession numbers for new data are not publicly accessible before the expected online posting of the article, publication may be delayed; please contact ASM production staff immediately with the expected release date.

Publication Fees: For information on publication fees and which article types have charges, please visit our website. We have partnered with Copyright Clearance Center (CCC) to collect author charges. If fees apply to your paper, you will receive a message from no-reply@copyright.com with further instructions. For questions related to paying charges through RightsLink, please contact CCC at ASM_Support@copyright.com or toll free at +1-877-622-5543. CCC makes every attempt to respond to all emails within 24 hours.

ASM Membership: Corresponding authors may join or renew ASM membership to obtain discounts on publication fees. Need to upgrade your membership level? Please contact Customer Service at Service@asmusa.org.

Embargo Policy: A press release may be issued as soon as the manuscript is posted on the mSystems Latest Articles webpage. The corresponding author will receive an email with the subject line "ASM Journals Author Services Notification" when the article is available online.

Cover Image Submissions: If you would like to submit a potential Cover Image, please email a file and a short legend to msystems@asmusa.org. Please note that we can only consider images that (i) the authors created or own and (ii) have not been previously published. By submitting, you agree that the image can be used under the same terms as the published article. Image File requirements: TIF/EPS, 7.5 inches wide by 8.25 inches tall (at least 2,250 pixels wide by 2,475 pixels tall), minimum 300 dpi resolution (600 dpi preferred), RGB, and no figure elements, e.g., arrows or panel labels. The legend should be a short description of the image, 1-2 sentences recommended. Please download and use this interactive template in Adobe to ensure that your proposed cover image meets our size requirements (https://journals.asm.org/pb-assets/pdf-text-excel-files/ASM-Interactive-Sizing-Cover-Template-1715689791.pdf).

Thank you for submitting your paper to mSystems.

Sincerely,
Rupinder Kaur
Editor
mSystems

Reviewer #3 (Comments for the Author):

The authors have addressed my previous concerns through their revisions. I am satisfied with the modifications.

Reviewer #4 (Comments for the Author):

I believe this revision has addressed all the concerns of the reviewers.
